# Cracking the Valence Code: Patterned Facial Kinematics and Neural Signatures of Emotional Expressions in Mice

**DOI:** 10.1002/advs.202417156

**Published:** 2025-08-20

**Authors:** Yujia Chen, Ruiqing Hou, Zhinan Chen, Junli Lu, Si Chen, Shisheng Xiong, Jianfeng Feng, Trevor W. Robbins, Haitao Yan, Xiao Xiao

**Affiliations:** ^1^ Behavioral and Cognitive Neuroscience Center Institute of Science and Technology for Brain‐Inspired Intelligence MOE Frontiers Center for Brain Science Key Laboratory of Computational Neuroscience and Brain‐Inspired Intelligence Ministry of Education Department of Endocrinology Huadong Hospital Fudan University Shanghai 200433 China; ^2^ College of Future Information Technology Fudan University Shanghai 200433 China; ^3^ Department of Educational Psychology Faculty of Education The Chinese University of Hong Kong Shatin Hong Kong SAR 999077 China; ^4^ Department of Psychology Behavioural and Clinical Neuroscience Institute University of Cambridge Cambridge CB2 3EB UK; ^5^ State Key Laboratory of National Security Specially Needed Medicines Beijing 100039 China

**Keywords:** AI‐driven emotion recognition, mouse facial expressions, neural signatures in the VTA, optogenetic modulation, valence‐specific patterns

## Abstract

Despite advances in linking mouse facial expressions to emotional states, the specific facial features and neural signatures remain elusive. An artificial intelligence (AI)‐based framework that decodes mouse facial expressions is presented, revealing stable valence and arousal dimensions analogous to those described in human emotion models. Facial expressions emerge as robust indicators of positive and negative emotional responses, validated through pharmacological manipulations, while responses to hallucinogens highlight the potential of valence‐specific prototype modeling for interpreting previously uncharacterized emotional states. Using automated multikeypoint tracking, patterned facial kinematics that are consistent within the same emotional valence are identified. Ear dynamics, in particular, emerge as critical features, offering distinct and sensitive markers of subtle emotional distinctions. Neurocorrelational analyses and optogenetic inhibition targeting the ventral tegmental area further demonstrate the intricate link between facial expressions and valence‐specific neural activity in dopaminergic and GABAergic neurons. These findings establish a precise, high‐temporal‐resolution platform for objectively decoding murine emotional states, advancing the understanding of emotional processing mechanisms and informing the development of mood‐regulating therapies.

## Graphical abstract

An AI‐powered system decodes emotional valence from mouse facial expressions, identifying ear dynamics as key indicators. By integrating facial kinematics with synchronized neural recordings, this approach establishes a direct link between behavior and brain activity, advancing automated emotion recognition in animal models.

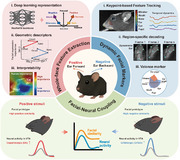

## 1 Introduction

Emotions are fundamental to physiological and psychological functions, shaping experience, guiding behavior, and supporting survival across species.^[^ ^1^, ^2^, ^3^ ^]^ While the definition of emotions remains debated, spanning views from consciously perceived feelings to adaptive survival mechanisms,^[^ ^4^, ^5^ ^]^ there is a widely accepted concept of basic emotions such as happiness, sadness, fear, and anger as evolutionarily ingrained and universally expressed.^[^ ^6^, ^7^ ^]^ These emotional states are associated with characteristic physiological and behavioral responses that serve critical adaptive functions.^[^ ^2^, ^8^, ^9^ ^]^ However, emotional experiences often extend beyond discrete categories.^[^ ^10^ ^]^ To address this complexity, a 2D model conceptualizes emotions along continuous axes of arousal (reflecting physiological and psychological activation) and valence (reflecting positive to negative evaluation), accommodating dynamic states that may not fall within basic emotional categories.^[^ ^10^, ^11^, ^12^ ^]^ For instance, fear and joy both involve relatively high arousal but differ in valence, with fear generally perceived as negative, while joy is associated with positive valence.^[^ ^11^ ^]^ From an evolutionary perspective, emotional behaviors are thought to be deeply conserved across species.^[^ ^4^, ^13^ ^]^ This homology supports the use of animal models to investigate the behavioral and neural mechanisms underlying emotional processing, with important implications for affective neuroscience and psychopathology research.^[^ ^13^, ^14^, ^15^ ^]^ However, determining how animals experience and express emotions remains a central challenge.

Facial expressions serve as a key to emotional communication, providing multicomponent signals that encode both discrete emotion categories (e.g., joy or anger) and continuous dimensions such as arousal and valence.^[^ ^12^, ^16^ ^]^ The Facial Action Coding System, developed from human studies, has been adapted to several animal species,^[^ ^17^ ^]^ including horses, primates, and dogs, facilitating systematic analysis of facial expressions across species.^[^ ^15^, ^18^, ^19^, ^20^, ^21^ ^]^ In small animals such as rodents, however, detecting subtle facial movements presents unique challenges. Early approaches primarily relied on subjective observations, limiting precision and reproducibility.^[^ ^22^ ^]^ The development of the Mouse Grimace Scale in 2010 provided a breakthrough in quantifying pain in mice through facial expressions.^[^ ^23^ ^]^ Recent advances in computer vision and machine learning have greatly enhanced the capacity to automatically detect and analyze subtle facial changes, enabling more precise insights into emotional states such as fear, pleasure, and pain.^[^ ^24^, ^25^, ^26^, ^27^, ^28^ ^]^

In mice that were well‐trained to display emotion‐related behaviors, neural activity can be correlated with emotion classification frameworks.^[^ ^5^, ^29^ ^]^ Emotional processing involves the coordinated activity of several key brain regions. For example, the lateral habenula (LHb) regulates aversion and negative affect, the amygdala modulates threat processing and valence, while the nucleus accumbens (NAc) integrates reward and motivational signals to drive goal‐directed behavior.^[^ ^5^, ^30^, ^31^, ^32^ ^]^ At the core of this network is the ventral tegmental area (VTA), which integrates inputs from multiple regions and orchestrates both reward and aversion,^[^ ^33^, ^34^, ^35^, ^36^, ^37^ ^]^ via its dopaminergic, GABAergic (GABA), and glutamatergic projections to regions such as the NAc, prefrontal cortex (PFC), and amygdala.^[^ ^33^, ^34^, ^35^, ^36^, ^37^ ^]^ The VTA's role in modulating both positive and negative emotional valence makes it a critical node for understanding the neural encoding of emotional states.^[^ ^34^ ^]^

In this study, we employed various positive and negative sensory stimuli to induce emotional state changes, generating measurable facial expressions. To improve objectivity and efficiency in measuring these emotions, we developed a comprehensive AI‐based framework that integrates multiple models for automated facial feature extraction and classification, prototype construction and validation, and dynamic keypoint tracking to identify facial components encoding valence‐specific emotional information. This approach not only refines the characterization of valence‐specific features but also enables the exploration of previously uncharacterized emotional shifts induced by hallucinogens. Furthermore, we combined neural recordings from the VTA with optogenetic inhibition to establish a direct link between facial expressions and valence modulation. Together, these findings underscore the pivotal role of the VTA in emotional processing and offer new insights into how emotional valence is encoded across both facial and neural domains.

## 2 Results

### 2.1 Facial Expressions of Mice Align with the Valence‐Arousal Model to Reveal Emotional States

**Figure 1:**
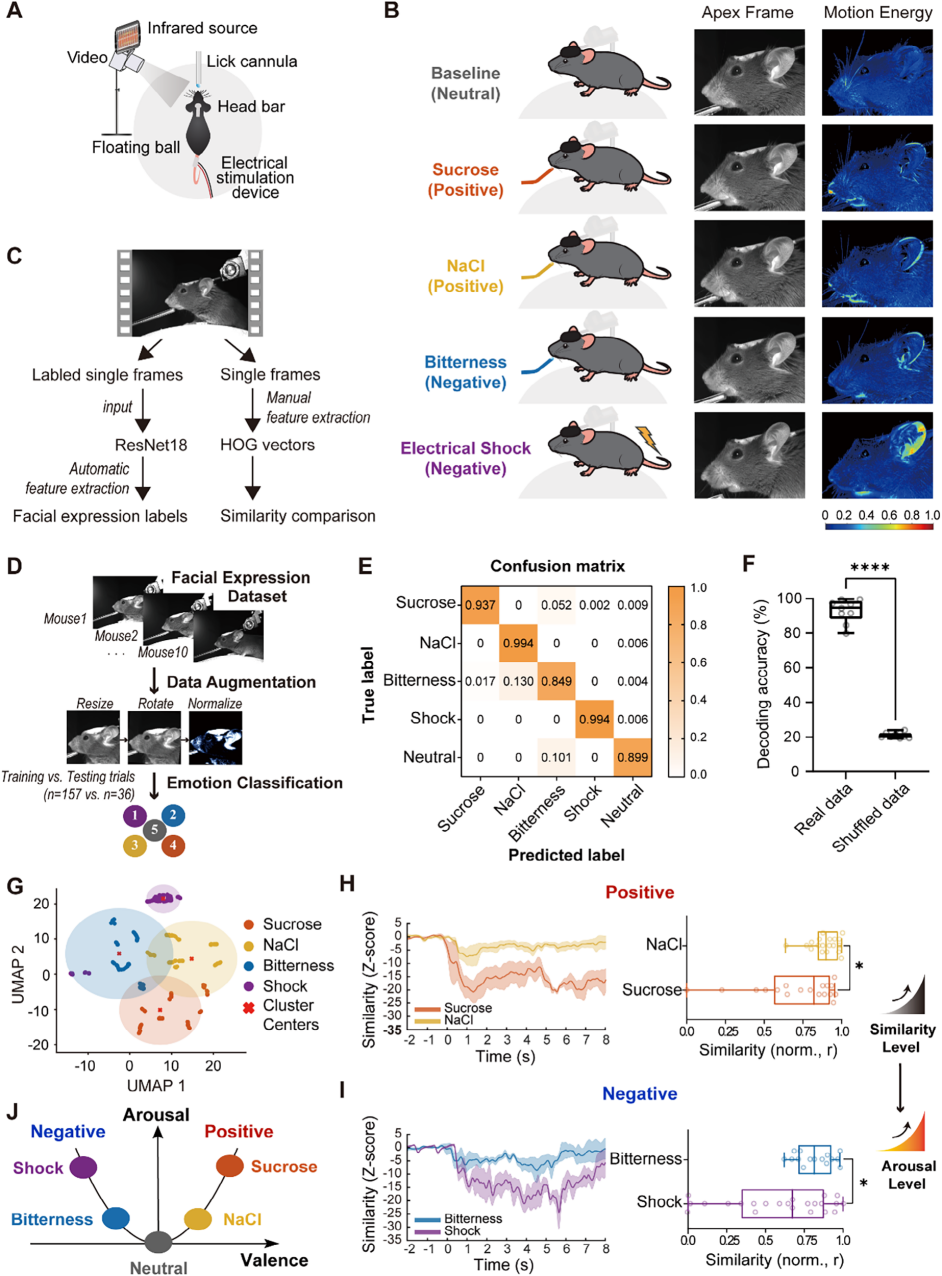
Facial expression analysis for emotion recognition in mice. A) Experimental setup for measuring facial expression changes in responding to different stimuli. B) Sample dataset overview. (left) The apex frame, captured during stimulation or neutral states, represents the moment of greatest facial difference in one exemplary mouse. (Right) Motion energy calculated from this exemplary mouse highlights areas of significant change in response to different stimuli or neutral states. C) Flow chart of emotional state assessment using manual and automatic feature extraction methods. D) Training the AI model based on ResNet18^[^ ^45^ ^]^ to classify the corresponding emotion states of the facial expression. E) Confusion matrix for classification of the test dataset collected from *N* = 9 mice. F) Mean classification accuracy of each mouse (*n* = 9) for real data compared to shuffled data in the independent test set. Each data point represents the average decoding accuracy for one mouse across all stimuli. Error bars indicate standard deviation across mice. ***p < 0.001, Welch's *t*‐test. G) UMAP visualization of facial expression values in response to all stimuli from an individual.^[^ ^47^ ^]^ Red marks indicate *k*‐means cluster centers, and shaded circles represent cluster boundaries defined by the maximum distance from these centers. H) (Left) Plot of changes in facial expression similarities (mean ± standard error of the mean (SEM)), computed using cosine similarity of HOG feature vectors, comparing neutral and poststimulation (sucrose and NaCl) states for an example animal. (Right) Quantifications for normalized similarities, data from *N* = 5 mice, and *n* = 18 trials for sucrose and *n* = 19 trials for NaCl. *p < 0.05, Mann–Whitney test. I) (Left) Plot of changes in facial expression similarities (mean ± SEM) comparing neutral and post‐stimulation (bitter and shock) states for an example animal. (Right) Quantifications for normalized similarities, data from *N* = 5 mice, and *n* = 15 trials for bitterness and *n* = 23 trials for shock. *p < 0.05, Mann–Whitney test. J) 2D circumplex model in mice. Four stimuli are represented by the following colors: sucrose (orange), NaCl (yellow), bitterness (blue), and electrical shock (purple).

To record facial expressions in mice, we used an infrared camera with head‐restrained, unanesthetized mice running freely on an air‐cushioned spherical treadmill (**Figure** **1**A). In a stimulus‐response paradigm, the mice were exposed to four sensory stimuli known to induce emotional responses (Figure 1B and Figure S1A (Supporting Information)): sucrose and low concentrations of sodium chloride (NaCl) to evoke hedonic responses,^[^ ^38^, ^39^, ^40^, ^41^ ^]^ a bitter solution to elicit aversive taste responses,^[^ ^38^, ^42^ ^]^ and tail shock to induce a full‐body aversive startle response.^[^ ^43^, ^44^ ^]^ We employed two complementary feature extraction methods to analyze facial expressions (Figure 1C).

First, we applied an end‐to‐end deep residual network ResNet18^[^ ^45^ ^]^ to classify facial expressions across stimuli (Figure 1C). A dataset of 29 100 facial images from 10 mice, spanning 157 trials covering neutral (baseline) and stimulus‐evoked expressions, was generated for model training (Figure 1C). Model performance was validated on an independent test dataset derived from 9 mice (one trial per stimulus per mouse, excluded from training; Figure 1D). The confusion matrix confirmed accurate classification across the four stimuli, with only minor misclassification between NaCl and bitterness stimuli (Figure 1E and Figure S1B (Supporting Information)). The model achieved a cross‐mouse decoding accuracy of 93.02 ± 2.26%, significantly outperforming the 21.00 ± 0.53% accuracy obtained with randomly shuffled data (Figure 1F). These results indicate that mice exhibit distinct, stimulus‐specific facial expressions corresponding to different emotional states, which can be accurately distinguished by our end‐to‐end approach, consistent with Dolensek et al.^[^ ^26^ ^]^

Beyond supervised classification, we further examined whether distinct emotional expressions could be detected using interpretable hand‐crafted features. For each stimulus, an apex frame—the most distinct and representative facial expression—was identified using Histogram‐of‐Oriented‐Gradients (HOG)‐^[^ ^46^ ^]^ based cosine similarity. HOG descriptors encode local edge orientation patterns and facial shapes, preserving the spatial configuration of facial features. The apex was determined via a two‐step process: first, the 10 frames most similar to other frames within the same stimulus condition were identified, representing the typical expression pattern; then, the frame least similar to baseline frames was selected, ensuring maximal contrast with the neutral state. Facial motion energy analysis across the 5 s stimulation windows revealed stimulus‐specific facial dynamics (Figure 1B).

Next, we assessed whether facial expressions formed distinct patterns in unsupervised feature space. HOG vectors from all frames were input into Uniform Manifold Approximation and Projection (UMAP),^[^ ^47^ ^]^ which revealed clear stimulus‐ and valence‐dependent clustering (Figure 1G and Figure S1D (Supporting Information)). Notably, stimuli of the same valence tended to cluster together: positive stimuli (sucrose and NaCl) were grouped closer to each other, while negative stimuli (tail shock and bitter solution) also formed a closer cluster (Figure 1G and Figure S1D (Supporting Information)). Importantly, mice receiving intraoral solutions via cheek fistula^[^ ^48^, ^49^ ^]^ exhibited similar clustering patterns (Figure S1C and Videos S1 and S2, Supporting Information), suggesting that oral movements alone during tastant delivery were unlikely to account for the observed separation in UMAP spaces (Figure S1D, Supporting Information). These results demonstrate that mouse facial expressions reliably encode valence information.

To examine arousal differences within valence groups, a similarity analysis was conducted using HOG vectors, comparing facial movements before and after stimulation. For positive emotions, sucrose exposure resulted in a lower trough similarity of facial expressions compared to NaCl, indicating stronger arousal (Figure 1H). Similarly, tail shock produced a more pronounced effect on facial expression similarity than the bitter solution within the negative valence group, reflecting a stronger aversive reaction (Figure 1I). Collectively, these results support that facial expressions of mice align with the valence–arousal model, effectively reflecting the 2D of emotional responses (Figure 1J).

### 2.2 Cross‐Mouse Generalization of Valence‐Specific Facial Expression Prototypes

**Figure 2:**
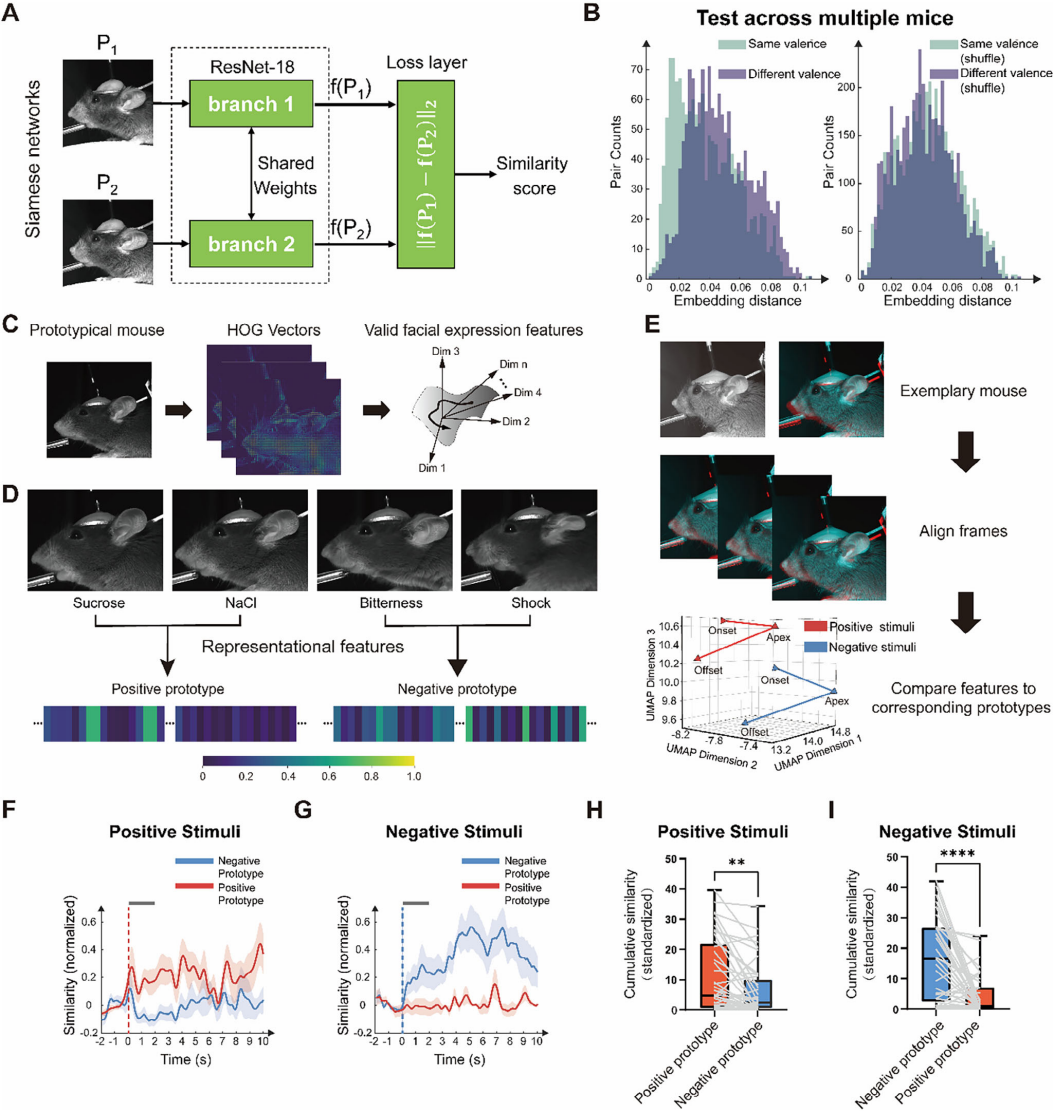
Representative valence‐based models of emotional facial expressions. A) Flow chart of the Siamese network^[^ ^52^ ^]^ architecture based on ResNet18 for valence‐specific facial expression analysis. B) (Left) Distributions of embedding distances for facial expressions in same‐valence pairs (green) and different‐valence pairs (purple) from *N* = 5 mice. (Right) Distributions with shuffled pair labels. Same‐valence pairs were significantly more tightly clustered than different‐valence pairs (**p < 0.001, two‐sided Welch's *t*‐test). C) Schematic for the feature extraction of facial expressions. D) Illustration of the most representative frames in four categories from a new mouse and the composition of valence‐specific facial features. The representational features, created as positive and negative prototypes, are perceived to convey emotional valence information. E) The illustration shows facial expression trajectories from a single positive and a single negative trial of one mouse, projected into the HOG–UMAP space at three time points (onset, apex, and offset), which were subsequently compared to the valence prototypes. F) Similarities of facial expressions for positive stimuli (*n* = 6 trials, 3 trials for sucrose and 3 trials for NaCl) in an individual mouse to the valence prototype. Line and shaded area are mean ± SEM. G) Same as (F), but for negative stimuli (*n* = 6 trials, 3 trials for bitterness and 3 trials for shock). H) Quantifications of cumulative similarities, based on data from *N* = 7 mice for positive stimuli (*n* = 45 trials, 25 trials for sucrose and 20 trials for NaCl), which are here generically labeled as red for results compared to the positive prototype and blue for results compared to the negative prototype. **p < 0.01, Wilcoxon signed‐rank test (paired). I) Same as (H), but for negative stimuli (*n* = 40 trials, 19 trials for bitterness and 21 trials for shock). ****p < 0.0001, Wilcoxon signed‐rank test (paired).

To investigate whether valence‐specific facial expression representations are not only present but also generalize across individual mice, a Siamese network based on ResNet18 was used to map orofacial images into a shared feature space. This data‐driven approach allowed the model to learn valence‐related patterns directly from the images without relying on predefined features (**Figure** **2**A).^[^ ^50^, ^51^, ^52^ ^]^ For training this model, we constructed a dataset from 13 mice using a novel matching strategy (Figure S2A, Supporting Information), labeling facial expression pairs as “different valences” when drawn from the same mouse but opposite valences, and as “same valence” when drawn from different mice but sharing the same valence. This approach ensured that the network focused on valence‐related features rather than individual‐specific features, enhancing its ability to distinguish between different valences based on essential features. After minimizing the contrastive loss, same valence pairs converged in the embedding space while different valence pairs dispersed, allowing the model to perform well on untrained mice without distinct label assignments. Embedding distances showed significant group‐level and individual‐level separation (Figure 2B and Figure S2B (Supporting Information)), indicating that the Siamese network successfully captured valence‐specific features of facial expressions consistent across mice. To visualize which facial regions contributed most to valence‐based similarity in the Siamese network, we applied a Grad‐CAM‐based visualization approach adapted for contrastive learning (Figure S2C (Supporting Information)).^[^ ^53^, ^54^, ^55^, ^56^ ^]^ Ears, mouth, and snout were consistently emphasized in similarity judgments, while dissimilarity often localized to ear and whisker regions, highlighting region‐specific roles in valence‐based similarity encoding. This finding supports the presence of consistent representations of positive and negative valence that extend across individual mice, suggesting a shared structural basis in how facial expressions encode emotional states.

Building on prior studies that emphasize the critical role of facial appearance, shape, and texture in mice, and that the effectiveness HOG in capturing such structural features,^[^ ^26^, ^27^, ^57^ ^]^ we next asked whether valance‐specific facial expression could also be detected using this interpretable, hand‐crafted descriptors. We used HOG descriptors to extract high‐dimensional feature vectors for each expression (Figure 2C) and averaged the most representative frames with the same valence into single HOG vectors, generating a positive prototype and a negative prototype (Figure 2D). These prototypes exhibited clear differences in feature space between positive and negative facial expressions. To test whether the valence prototype represented by HOG vectors captured meaningful emotional information, we compared the facial expressions from additional trials to these prototypes within the HOG‐based embedding space (Figure 2E). This approach allowed visualization of trial‐wise expression dynamics and their separation by valence. Prior to this comparison, the face region was prealigned to a prototypical mouse template, ensuring precise tracking of emotional‐valence‐related characteristics (Figure 2E). Under positive stimuli, facial expressions showed significantly higher similarity to the positive prototype than to the negative prototype (Figure 2F,H), whereas expressions induced by negative stimuli more closely matched the negative prototype (Figure 2G,I). These findings demonstrate that HOG‐based prototypes effectively capture valence‐specific facial representations, offering a quantitative framework for analyzing emotional valence.

### 2.3 Facial Expressions as Real‐Time Indicators of Drug‐Induced Emotional States

**Figure 3:**
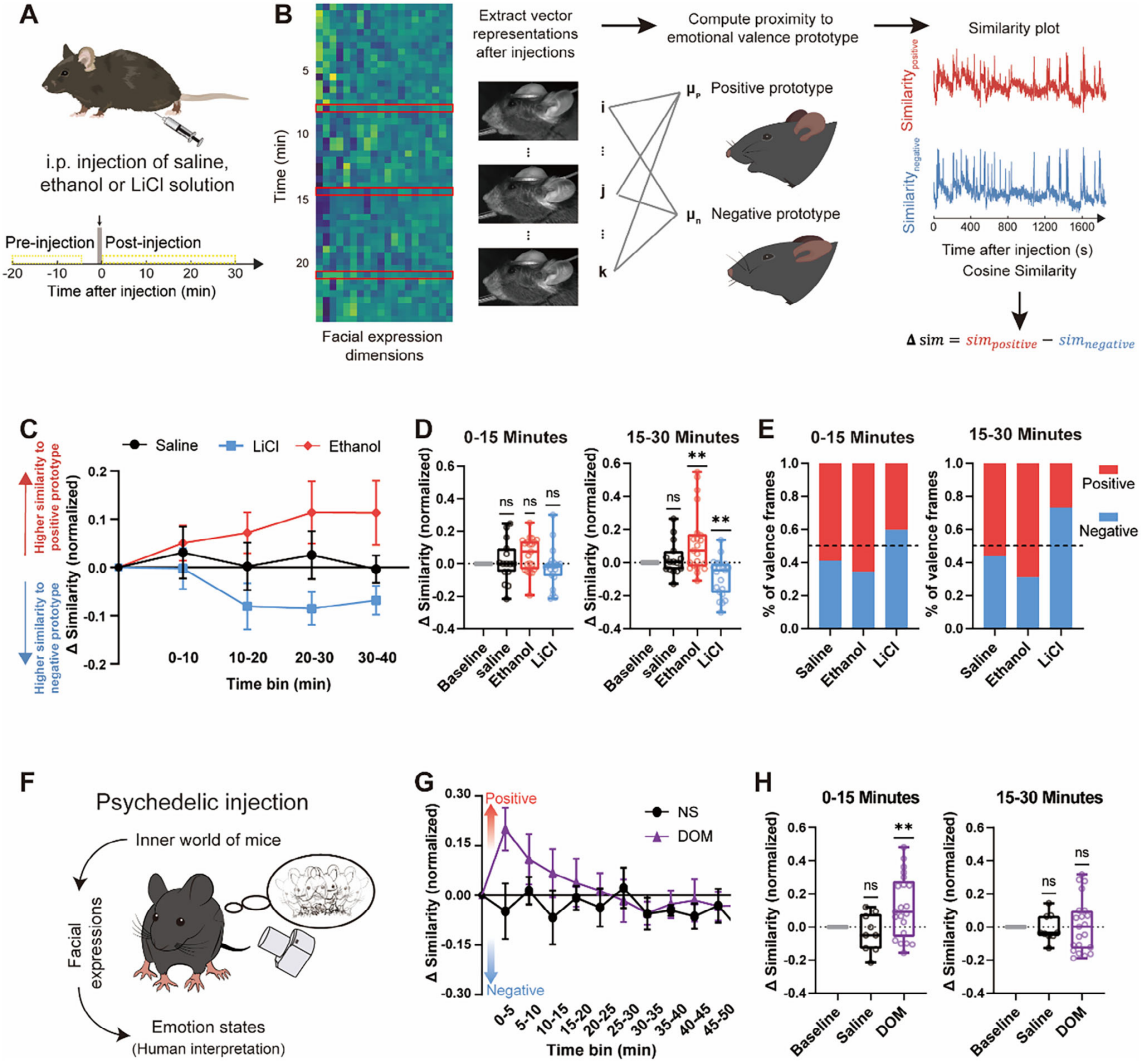
Assessment of drug‐induced variability of emotional facial expressions in mice. A) Schematic for the experimental timeline for drug‐induced facial expression recordings. Facial expressions were analyzed from 20 min before intraperitoneal (i.p.) injection to 30 min after the injection. B) Procedure for valence assessment of drug‐induced facial expressions. C) Time course of facial expressions induced by saline (*N* = 5 mice, black), ethanol (*N* = 7 mice, red), or LiCl (*N* = 7 mice, blue) intraperitoneal injections. The data points greater than 0 indicate that real‐time facial expressions show higher similarity to the positive prototype, whereas data points less than 0 suggest that real‐time facial expressions are more similar to the negative prototype. Data are mean ± SEM. D) Quantifications of normalized similarity differences: 0–15 min (left) and 15–30 min (right) postinjection. Data for saline (*N* = 5 mice, black), ethanol (*N* = 7 mice, red), and LiCl (*N* = 7 mice, blue). **p < 0.01, one‐sample *t*‐test with Bonferroni correction. E) Proportions of calculated positive and negative facial expressions: 0–15 min (left) and 15–30 min (right) postinjection. Data for saline (*N* = 5 mice, black), ethanol (*N* = 7 mice, red), and LiCl (N = 7 mice, blue). F) Illustration of internal emotional states through facial expression recording following injection. G) Time course of facial expressions induced by saline (*N* = 3 mice, black) and DOM (*N* = 7 mice, purple) injections. Data are mean ± SEM. H) Quantifications of normalized similarity differences: 0–15 min (left) and 15–30 min (right) postinjection. Data for saline (*N* = 3 mice, black) and DOM (*N* = 7 mice, purple). **p < 0.01, one‐sample *t*‐test with Bonferroni correction.

Building on these results, we extended our framework to assess drug‐induced emotional responses using saline, ethanol, and lithium chloride (LiCl) administration (**Figure** **3**A). Whole‐face motion energy analysis revealed distinct differences in facial movement dynamics across drug conditions (Figure S3B, Supporting Information). For detailed analysis, each frame from a single experiment was converted into high‐dimensional HOG features (Figure 3B), and the dynamics of facial expressions over time were quantified by comparing frame‐wise similarity to the valence prototypes. Following ethanol administration, facial expressions increasingly resembled the positive prototype, while LiCl‐treated mice aligned more closely with the negative prototype. Saline‐injected mice displayed minimal fluctuations between prototypes, indicating a neutral profile (Figure 3C). From 15 min onward, differences between groups became more pronounced: ethanol‐treated mice showing significant increases in similarity to the positive prototypes, while LiCl‐treated mice showing enhanced similarity to negative emotion prototypes (Figure 3D). We further quantified valence shifts by calculating the proportion of frames resembling each prototype, expressed as the ratio of positive‐prototype‐matching frames to negative‐prototype‐matching frames (Figure 3E). During 0–15 min, positive frames exceeded negative by 31.1% in the ethanol group, while negative frames exceeded positive by 19.5% in the LiCl group. These differences became more pronounced between 15 and 30 min. The saline group remained largely stable (Figure 3E). These results are consistent with previous studies showing that ethanol and LiCl induce positive and negative emotional states, respectively, reflected in facial expressions resembling the corresponding affective prototypes.^[^ ^58^, ^59^, ^60^, ^61^, ^62^, ^63^, ^64^ ^]^

We next examined the effects of the hallucinogen 2,5‐dimethoxy‐4‐methylamphetamine (DOM) (Figure 3F), known for its profound impact on emotion, perception, and cognition.^[^ ^65^, ^66^ ^]^ DOM induced a rapid onset of emotional response, with facial expressions shifting significantly toward the positive prototype within the first 15 min (Figure 3G), followed by a return to baseline between 15 and 30 min (Figure 3H). Motion energy analysis showed a parallel reduction in overall facial motion during this initial period, which gradually recovered thereafter (Figure S3B, Supporting Information). This temporal profile closely mirrors previous observations of hallucinogen‐induced head‐twitch responses, typically peaking at 6–12 min postadministration.^[^ ^67^, ^68^, ^69^ ^]^ Importantly, our findings provide novel evidence that this prominent motor activity reflects not merely stereotyped movements but is tightly linked to a transient positive emotional state, as captured by facial expression dynamics. Furthermore, these drug‐induced emotional shifts exhibit distinct short‐timescale dynamics (Figure S3B,C, Supporting Information), highlighting the sensitivity of real‐time facial expression analysis in capturing the transient emotional effects of psychedelics.

### 2.4 Patterned Facial Kinematics Reveal Interpretable Valence‐Specific Features

**Figure 4:**
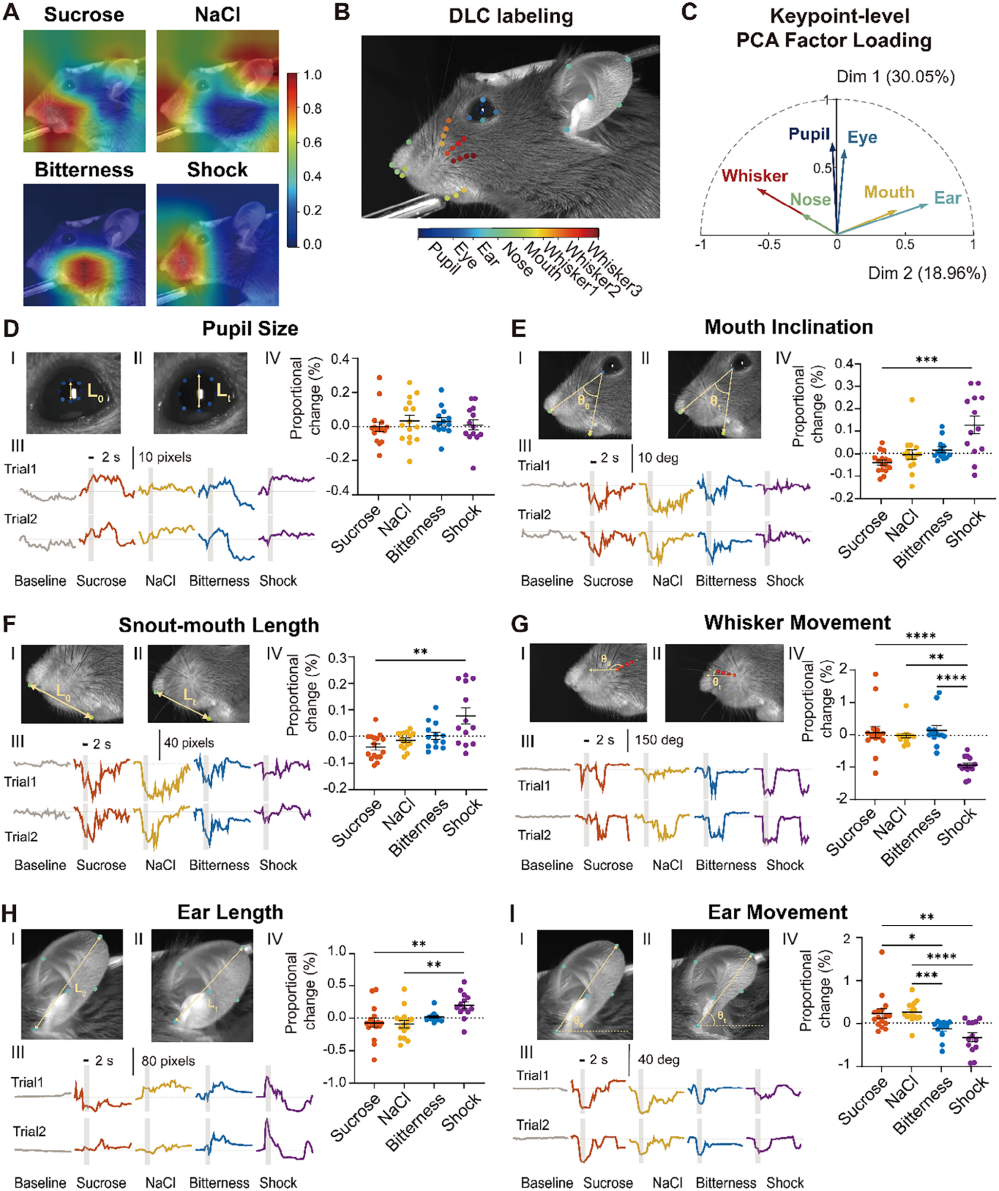
Keypoint tracking of feature regions in mice. A) Utilizing Grad‐CAM^[^ ^56^ ^]^ to highlight key regions focused on by ResNet18 during classification of emotional states. B) Full‐face tracking of 37 keypoints (8 for the pupils, 4 for the eyes, 5 for the ears, 5 for the nose, 3 for the mouth, and 12 for the whiskers) using DEEPLABCUT.^[^ ^70^ ^]^ C) Principal component analysis of keypoint patterns in mouse facial expressions across various stimuli (3420 frames [57 trials × 60 frames per trial] × 12 features [6 keypoints × (*x*, *y*)]). Six representative keypoints were selected based on maximal variance across trials, with one from each of the following facial regions: ear, whisker pad, snout, mouth, eye, and pupil. D) (Top left, I and II) Visualization of identified pupil size on the mouse profile. (Bottom left, III) Dynamic curves for the corresponding facial regions in a sample mouse under 2 repeated stimulations. (Right, IV) Quantification of peak proportional changes relative to baseline across *N* = 5 mice (*n* = 16 trials for sucrose, *n* = 15 trials for NaCl, *n* = 13 trials for bitterness, and *n* = 13 trials for shock), with adjustments made by subtracting the water session peak proportion. E) Same as (D), but for identified mouth inclination, illustrating changes in the angle of mouth tilt. F) Same as (D), but for identified snout–mouth length, illustrating the distance variation between the tip of the snout and bottom lip. G) Same as (D), but for identified whisker movement, illustrating the angle of whisker motion. H) Same as (D), but for identified ear length, illustrating the extension or contraction in the length of ear pavilion. I) Same as (D), but for identified ear movement, illustrating the rotation angle of the ear. The results are shown as individual data points with error bars, mean ± SEM. *p < 0.05, **p < 0.01, ***p < 0.001, ****p < 0.0001, Kruskal–Wallis ANOVA followed by Dunn's post‐hoc test.

To identify specific facial regions contributing to emotional expression, we applied Grad‐CAM to the ResNet18 classifier, generating heatmaps highlighting key regions (**Figure** **4**A). The model predominantly relied on the oral region, ears, and whiskers for classification, with distinct focal areas for each stimulus. To incorporate geometric morphometrics into the analysis, we conducted full‐face keypoint tracking using DeepLabCut^[^ ^70^ ^]^ with 37 keypoints, allowing for a more precise evaluation of facial movements across stimuli (Figure 4B). Principal Component Analysis (PCA) reduced the dimensionality of the keypoint dataset, revealing coordinated facial motions: the first component (Dim1; 30.05%) reflected global movement across multiple features; the second component (Dim2; 18.96%) was dominated by ear movements (Figure 4C). All keypoint data were *z*‐scored to control for interregional amplitude differences prior to PCA.

Next, we quantified the dynamic changes across key facial features: pupil size, mouth inclination, snout–mouth length, whisker movement, ear length, and ear rotation (Figure 4D–I). While response latencies were consistent across stimuli, they varied across features (Figure S4E–I, Supporting Information): whisker responses were the fastest, while pupil dilation lagged. Although pupil size was affected by all stimuli, no significant between‐condition differences were observed (Figure 4D). By contrast, mouth inclination, snout–mouth length, and whisker movement displayed significant valence‐dependent differences (Figure 4E–G), with the most robust distinctions observed in ear dynamics: ear length and rotation showed opposing trends between positive and negative stimuli (Figure 4H,I).

**Figure 5:**
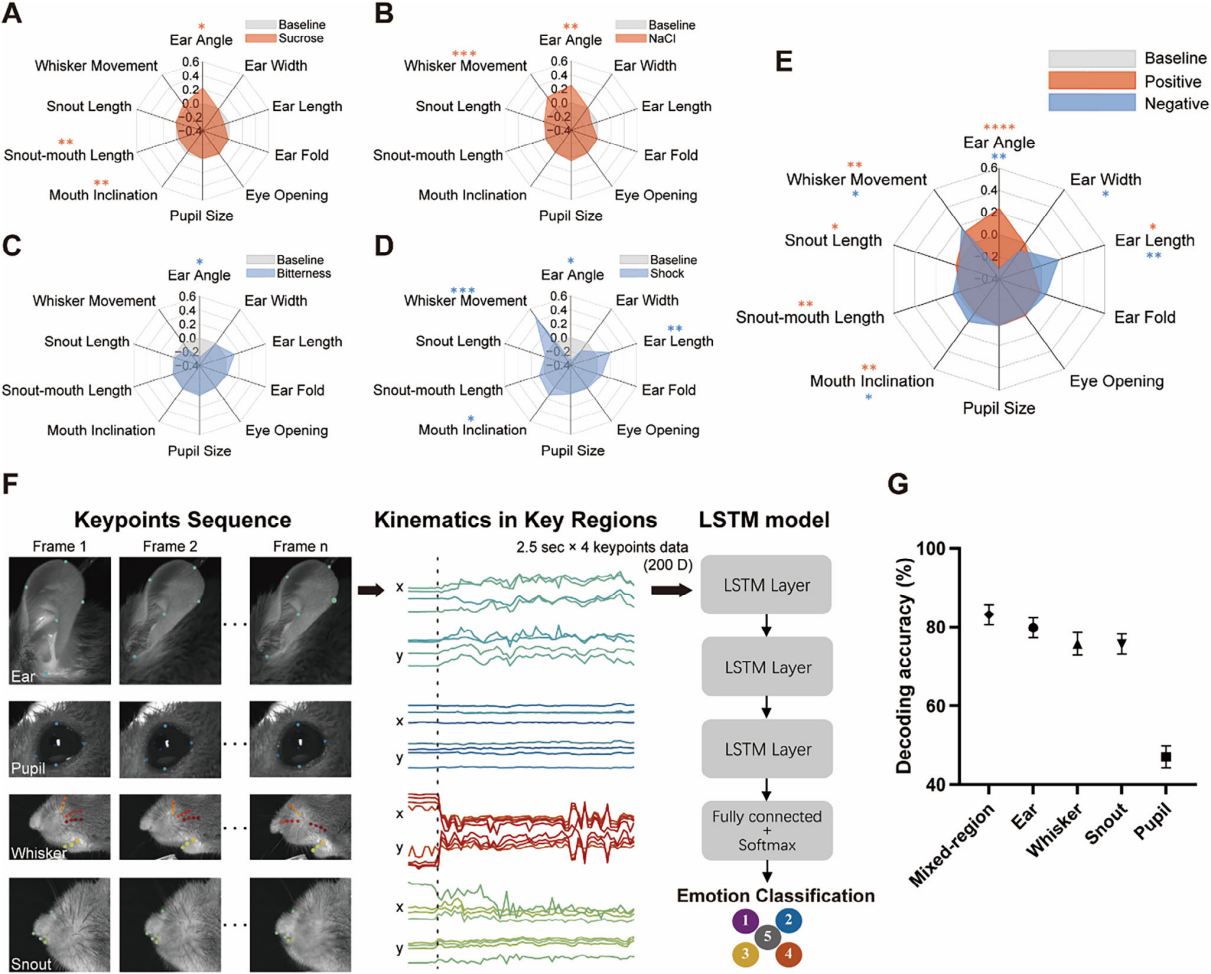
Consistent movements of facial regions in response to positive and negative emotion. A) Visual representation of the proportional changes from baseline in facial regions during sucrose stimulation across *N* = 5 mice (*n* = 16 trials for sucrose, *n* = 15 trials for NaCl, *n* = 13 trials for bitterness, and *n* = 13 trials for shock). Data are presented as mean values. *p < 0.05, **p < 0.01, one sample, two‐sided Wilcoxon test. B) Same as (A), but in response to NaCl. C) Same as (A), but in response to bitterness. D) Same as (A), but in response to shock. E) Visual representation of patterned valence‐based facial actions, merging positive stimuli (sucrose, NaCl) and negative stimuli (bitterness, shock). Each panel (A–D) presents results from separate trial sets without data overlap. E) Aggregated trials by valence for independent group‐level analysis. *p < 0.05, **p < 0.01, ****p < 0.0001, one sample, two‐sided Wilcoxon test. F) LSTM‐based decoding of emotional facial expressions from tracked keypoints. Representative facial keypoints were tracked over time in four regions: the ear, pupil, whisker, and snout (left). The *x*/*y* coordinates of these keypoints were converted into spatiotemporal trajectories (middle), forming a 200D input to a multilayer LSTM network (right). This feature vector was constructed from 4 region‐specific keypoints × 2 spatial axes × 2.5 s × 10 frames s^−1^. G) Classification performance was assessed using LSTM models trained on trajectories from each individual facial region (ear, whisker, snout, or pupil), with each region represented by four keypoints. For comparison, the “mixed‐region” condition used four keypoints randomly selected from across all regions. Bars indicate mean decoding accuracy ± SEM across cross‐validation folds.

By integrating these geometric measures, we identified coordinated facial feature patterns that exhibited strong within‐valence similarity and distinct topological structures across stimuli (**Figure** **5**A–D). Ear angle deviated significantly from baseline under all conditions, with sucrose and NaCl inducing similar shifts, while bitterness and shock elicited opposing changes. Positive stimuli formed a stone‐like pattern (Figure 5A,B), whereas negative stimuli resembled a heart‐like structure (Figure 5C,D). When combining parameters across valences, opposing trends in ear angle and mouth inclination became more prominent, consistent with previous findings (Figure 5E). This patterned facial action kinematics clearly reflected valence‐specific emotional expressions. To rule out the possibility that facial expression differences were confounded by stimulus‐induced running behavior, we analyzed high‐motion trials from three mice that exhibited running following both positive (sucrose or NaCl) and negative (shock) stimuli. Despite similar overall motion energy and whisker displacement across conditions, ear posture remained distinct: forward ear angles during positive trials and retracted ears during shocks (Figure S5A,B, Supporting Information). UMAP projections based on HOG features further confirmed valence‐specific separation even under intense movement (Figure S5C, Supporting Information), supporting the robustness of ear movements as emotional indicators.

We further assessed region‐specific contributions to emotion decoding using a time‐resolved Long‐Short‐Term‐Memory (LSTM)‐based classifier based on DeepLabCut‐derived keypoints (Figure 5F,G).^[^ ^71^ ^]^ For each of four representative regions (ear, whisker, snout, pupil), sequential (*x*, *y*) coordinates over a 2.5 s window (0.5 s pre‐ to 2 s poststimulus) were extracted. The ear region alone achieved the highest decoding accuracy (79.85 ± 7.58%) among all individual regions, nearly matching the mixed‐region model that incorporated keypoints from all regions (83.18 ± 7.47%). While the whisker (75.76 ± 8.56%) and snout (75.68 ± 7.73%) regions also contributed substantially, the pupil region (47.05 ± 8.35%) performed the poorest (Figure 5F,G). All regions showed improved classification relative to single‐frame models, highlighting the importance of temporal dynamics. To complement these analyses, we performed an occlusion sensitivity test using the ResNet18 classifier. Occluding the ear region led to the greatest decline in classification accuracy, followed by the mouth occlusion, whereas occlusion of the eyes or background had minimal effect (Figure S5D,E, Supporting Information). Together, the LSTM and occlusion analyses confirm that ear movements provide temporally rich, dominant signals for decoding emotional state in mice.

### 2.5 VTA Modulation of Emotional Valence Shapes Facial Expression Dynamics

**Figure 6:**
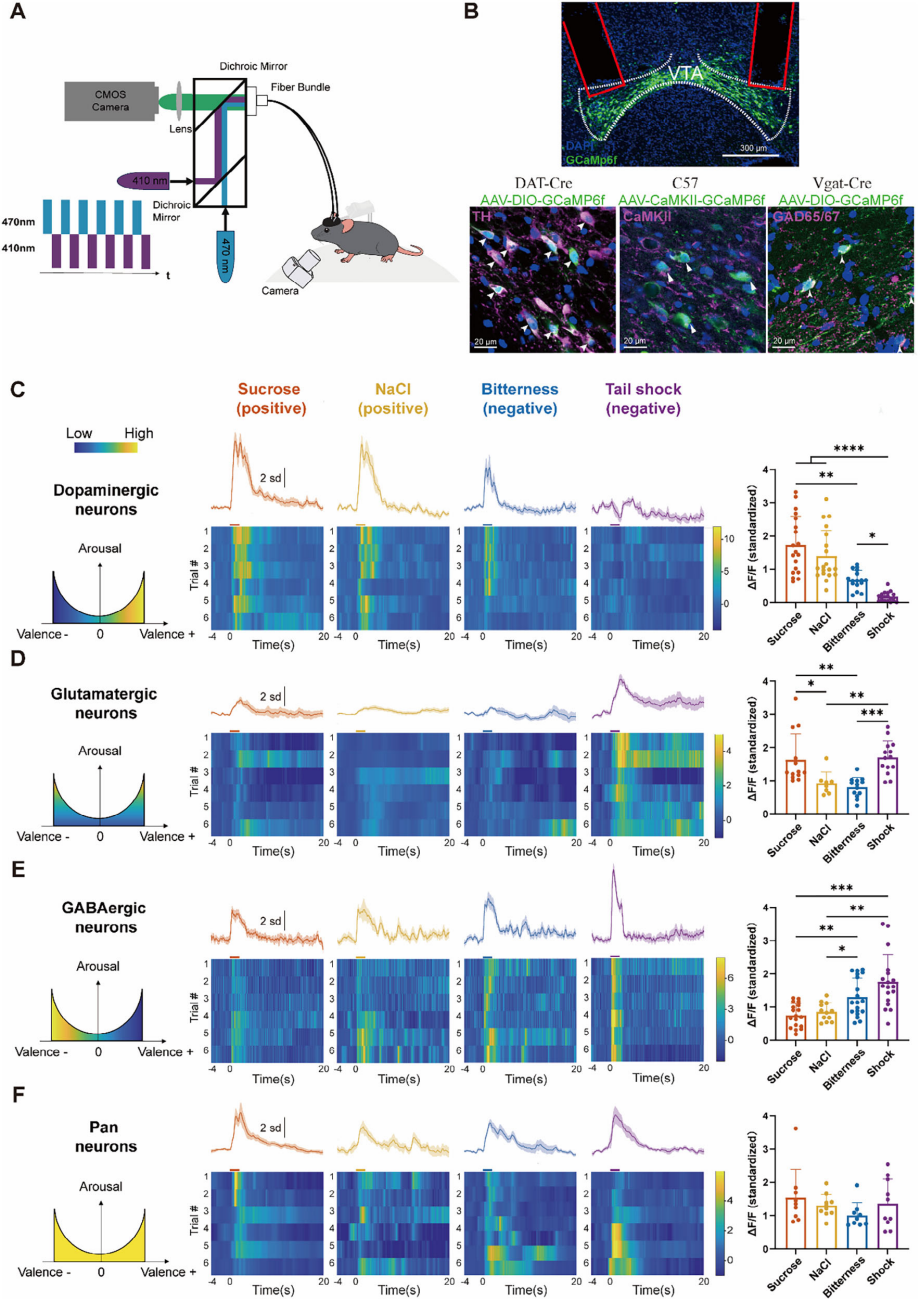
Calcium response patterns of dopaminergic, glutamatergic, GABAergic, and pan neurons in the VTA. A) Schematic diagram of experiment setup for fiber photometry and facial recording. B) Schematic illustrating the virus injection site, and optic fiber placement. (Top) Green fluorescence showing GCaMP6f expression colocalized with 4’,6‐diamidino‐2‐phenylindole (DAPI) in the VTA, alongside the placement of the optic fiber. Scale bars, 300 µm. (Bottom) GCaMP6f expression (green) in VTA dopaminergic neurons (tyrosine hydroxylase (TH), magenta), glutamatergic neurons (Calcium–calmodulin (CaM)‐dependent protein kinase II (CaMKII), magenta), and GABAergic neurons (glutamic acid decarboxylase 65/67 (GAD65/67), magenta), merged with DAPI (blue). Scale bars, 20 µm. Across slices, viral expression was highly specific, with 82.5 ± 6.2% of GCaMP6f‐expressing neurons also positive for TH, 91.7 ± 1.2% for CaMKII, and 88.5 ± 2.4% for GAD65/67. A substantial proportion of target neurons was captured in recordings, with 76.5 ± 10.9% of TH‐positive, 76.2 ± 10.4% of CaMKII‐positive, and 80.4 ± 9.6% of GAD65/67‐positive neurons expressing GCaMP6f (*n* = 4 slices from 2 mice per group). C) (Left) U‐shaped curve model based on the valence‐arousal framework, illustrating VTA^DA^ calcium signals in response to varying emotional stimuli. (Middle) Representative dopamine transporter–Cre (DAT‐Cre) mouse displaying the average profile (mean ± standard deviation (SD)) and heatmap illustration of VTA^DA^ Ca^2+^ signals across *n* = 6 trials in response to four stimuli: sucrose, NaCl, bitterness, and shock. The color bar denotes the 2 s stimulus duration. (Right) Quantification of peak Ca^2+^ response variations in VTA^DA^ neurons from *N* = 3 DAT‐Cre mice (*n* = 19 trials for sucrose, *n* = 19 trials for NaCl, *n* = 16 trials for bitterness, and *n* = 19 trials for shock). *p < 0.05, **p < 0.01, ***p < 0.001, ****p < 0.0001, Kruskal–Wallis ANOVA followed by Dunn's post‐hoc test. D) (Left, middle) Same as (C), but for VTA^GLU^ neurons in C57BL/6J mice. (Right) Quantification of variations in peak Ca^2+^ responses in VTA^GLU^ neurons from *N* = 3 C57 mice (*n* = 13 trials for sucrose, *n* = 8 trials for NaCl, *n* = 13 trials for bitterness, and *n* = 14 trials for shock). E) (Left, middle) Same as (C), but for VTA^GABA^ neurons in vesicular GABA transporter–Cre (VGAT‐Cre) mice. (Right) Quantification of variations in peak Ca^2+^ responses in VTA^GABA^ neurons from *N* = 3 VGAT‐Cre mice (*n* = 18 trials for sucrose, *n* = 12 trials for NaCl, *n* = 19 trials for bitterness, and *n* = 18 trials for shock). F) (Left, middle) Same as (C), but for pan‐VTA neurons, representing overall Ca^2+^ responses across all neuron types. (Right) Quantification of variations in peak Ca^2+^ responses in pan‐VTA from *N* = 3 C57 mice (*n* = 9 trials for sucrose, *n* = 10 trials for NaCl, *n* = 9 trials for bitterness, and *n* = 10 trials for shock).

To link facial expressions with neural activity, we employed fiber photometry to record calcium dynamics from dopaminergic (DA), glutamatergic (Glu), and GABA neurons in the VTA while simultaneously recording facial expressions during emotional stimulation (**Figure** **6**A,B). VTA^DA^ neurons exhibited robust calcium peaks in response to positive stimuli (sucrose or NaCl), but much weaker responses to negative stimuli (bitterness or shock), consistent with their established role in encoding rewarding or positively valanced contexts (Figure 6C).^[^ ^72^ ^]^ By contrast, VTA^GABA^ neurons responded more strongly to negative stimuli, with significantly higher calcium peaks than those evoked by positive stimuli, supporting their role in processing aversive emotional states (Figure 6E).^[^ ^73^ ^]^ VTA^Glu^ neurons responded to high‐arousal stimuli (sucrose, shock), suggesting encoding of motivational salience rather than valence (Figure 6D).^[^ ^74^ ^]^ No significant differences were observed in pan‐neuronal activity across stimuli (Figure 6F).

**Figure 7:**
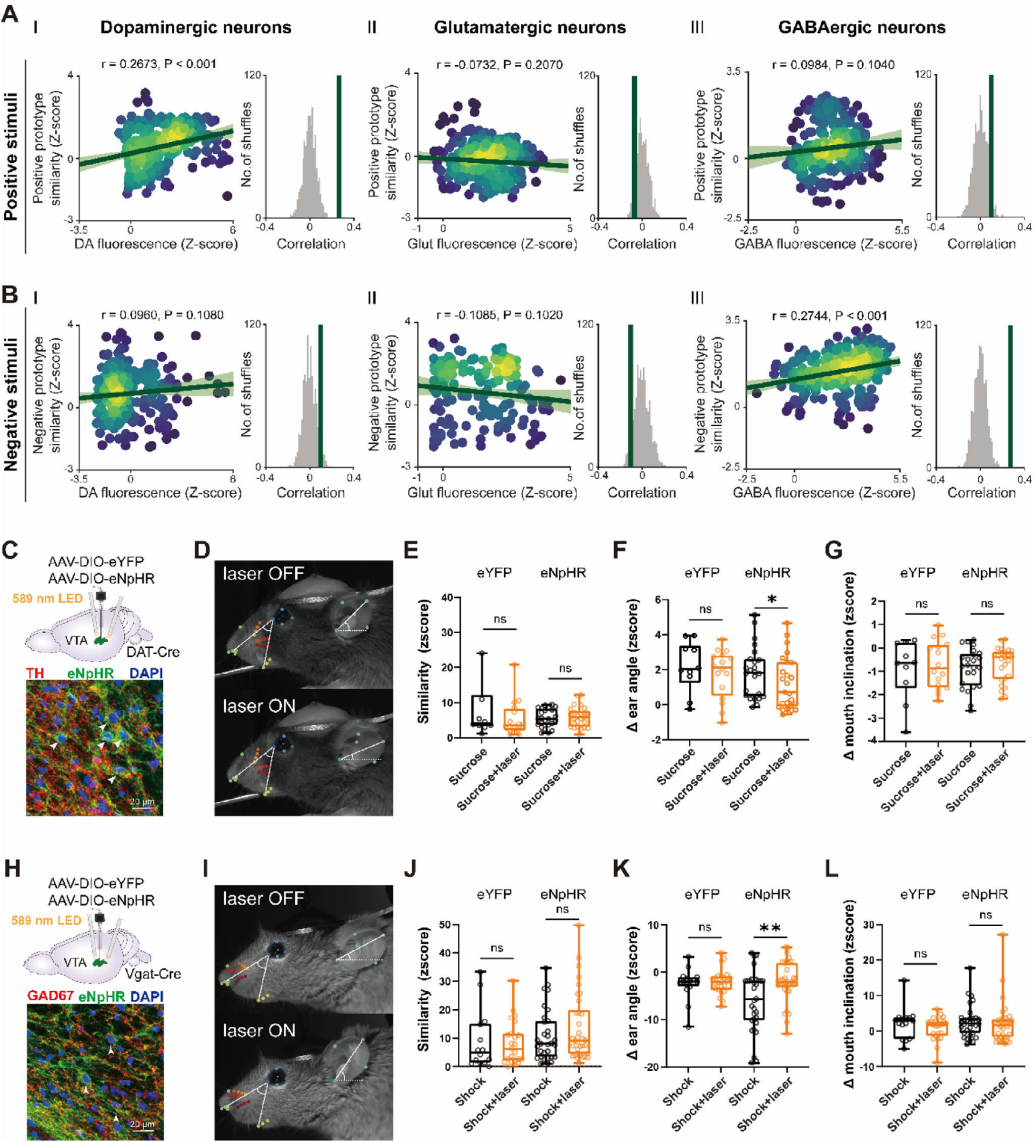
Optogenetic inhibition of VTA induced variations of facial expressions. A) The correlation between neural responses and facial movements: (left) linear regressions (green line, shaded 95% confidence interval) between *z*‐scored Ca^2+^ fluorescence signals and facial similarity to positive emotional prototypes during positive stimuli for three neuron types: (I) dopaminergic, (II) glutamatergic, and (III) GABAergic neurons. Each data point represents a single time point within the 2 s window following stimulus onset, with neural and facial data aligned at 15 Hz. (Right) The observed Spearman correlation (green line) is compared to a distribution generated by repeatedly shuffling data labels and recalculating the correlation after each shuffle. *p*‐values are estimated from a one‐sided shuffle test. B) Same as (A), but showing the linear regressions between Ca^2+^ fluorescence signals and facial similarity to negative emotional prototypes during negative stimuli. C) (Top): Schematic showing AAV injection and optogenetic inhibition site in the VTA of DAT‐Cre mice. (Bottom): Confocal image showing co‐localization of enhanced Natronomonas pharaonis Halorhodopsin (eNpHR) virus expression (Green) in TH‐immunopositive neurons (red) in the VTA of DAT‐Cre mouse. DAPI (blue) marks nuclei. In DAT‐Cre mice, 94.8 ± 3.9% of NpHR‐expressing neurons were TH‐positive, and 73.7 ± 3.2% of TH‐positive neurons expressed NpHR (*n* = 4 slices from 2 mice per group). D) Example images of facial expression in a DAT‐Cre mouse under laser‐Off (top) and laser‐On (bottom, with 20 Hz optical stimulation of VTA^DA^ neurons) during sucrose stimuli. E) Quantification of *z*‐score similarities in facial expressions for the enhanced yellow fluorescent protein (eYFP) group (*N* = 3 DAT‐Cre mice, *n* = 12 trials for laser‐Off, *n* = 18 trials for laser‐On) and the eNpHR group (*N* = 2 DAT‐Cre mice, *n* = 24 trials for laser‐Off, *n* = 25 trials for laser‐On). *p < 0.05, Mann–Whitney test. F) Same as (E), but quantification of peak ear angle changes from baseline *z*‐scored data. G) Same as (E), but quantification of peak mouth inclination changes from baseline *z*‐scored data. H) Same as (C), but GAD65/67 (red) labels GABAergic neurons in Vgat‐Cre mice. In Vgat‐Cre mice, 91.8 ± 5.2% of NpHR‐expressing neurons were GAD65/67‐positive, and 78.1 ± 7.0% of GAD65/67‐positive neurons expressed NpHR (*n* = 4 slices from 2 mice per group). I) Same as (D), but in a Vgat‐Cre mouse with 20 Hz optical stimulation of VTA^GABA^ neurons during shock stimuli. J) Quantification of *z*‐score similarities in facial expressions for the eYFP group (*N* = 2 Vgat‐Cre mice, *n* = 14 trials for laser‐Off, *n* = 23 trials for laser‐On) and the eNpHR group (*N* = 4 Vgat‐Cre mice, *n* = 31 trials for laser‐Off, *n* = 34 trials for laser‐On). *p < 0.05, **p < 0.01, Mann–Whitney test. K) Same as (J), but quantification of peak ear angle changes from baseline *z*‐scored data. L) Same as (J), but quantification of peak mouth inclination changes from baseline *z*‐scored data.

Correlational analysis revealed valence‐specific relationships between VTA neural activity and facial expressions (**Figures** **7**A,B and S6A–C (Supporting Information)). VTA^DA^ activity positively correlated with similarity to positive facial prototypes during sucrose stimulation (Figure 7A,B(I) and Figure S6A (Supporting Information)), while VTA^GABA^ activity correlated with negative prototypes during shock (Figure 7A,B(III) and Figure S6C (Supporting Information)). VTA^Glu^ neurons displayed no significant correlations with facial valence metrics (Figure 7A,B(II) and Figure S6B (Supporting Information)). To directly test causal links between VTA activity and facial expressions, we applied optogenetic inhibition of VTA^DA^ and VTA^GABA^ neurons, the predominant neurons in the VTA,^[^ ^75^ ^]^ during emotional stimulation (Figure 7C–L).

Inhibiting VTA^DA^ neurons during positive stimuli did not significantly affect global facial similarity (Figure 7E and Figure S6D (Supporting Information)), but selectively reduced ear angle variation, flattening ear movements (Figure 7F and Figure S6D(II) and Video S3 (Supporting Information)). Mouth inclination remained unaffected (Figure 7G and Figure S6D(III) (Supporting Information)), suggesting regional specificity of DAergic modulation. Inhibiting VTA^GABA^ neurons during negative stimuli led to significant alterations in ear dynamics, shifting ear posture during aversive conditions (Figure 7K and Figure S6D(I) and Video S4 (Supporting Information)), while overall facial configuration and mouth movements were unchanged (Figure 7J,L and Figure S6E(I, III) (Supporting Information)). Control animals (enhanced yellow fluorescent protein, eYFP) showed no effects, confirming specificity. In the absence of external stimuli, inhibition of VTA^DA^ or VTA^GABA^ neurons did not alter global or region‐specific facial expressions (Figure S6F,G, Supporting Information), confirming that the observed effects reflect modulation of stimulus‐evoked emotional responses. Overall, these findings suggest that ear movements are more sensitive to VTA neural inhibition, while mouth movements appear less responsive, potentially reflecting the specific neural circuits that modulate emotional states, leading to distinct changes in facial regions.

## 3 Discussion

In this study, we integrated high‐resolution facial expression analysis with neural activity measures to investigate emotional states in mice. Leveraging an end‐to‐end deep learning framework for automated facial feature extraction and classification, combined with pharmacological manipulations, neural activity monitoring, and optogenetic interventions, we characterized valence‐specific facial kinematics and their neural correlates. This comprehensive approach establishes facial expressions as dynamic, real‐time indicators of emotional states and provides mechanistic insights into the neural basis of emotional processing.

### 3.1 AI‐Assisted Decoding of Facial Expressions Reveals Interpretable Valence‐Specific Patterns

Emotions manifest as distinct facial expressions, reflecting internal affective states across species.^[^ ^10^, ^76^ ^]^ Our study demonstrates that, despite their relatively limited facial musculature compared to humans, mice exhibit complex and nuanced facial expressions encoding emotional states. By leveraging AI‐based models, we developed an end‐to‐end system capable of automatically extracting, characterizing, and classifying rodent‐specific facial expressions, minimizing reliance on subjective scoring and extensive preprocessing. This approach enabled sensitive, objective detection of emotional shifts, positioning facial expressions as powerful real‐time indicators of internal emotional processes.

However, the “black box” nature of advanced algorithms often raises concerns about interpretability and generalizability.^[^ ^77^ ^]^ To enhance model interpretability, we integrated Grad‐CAM into our model to visualize critical facial regions informing the network's decisions, confirming that biologically meaningful features such as ear positioning, mouth inclination, and whisker dynamics drive classification outputs.^[^ ^78^ ^]^ This transparency enhances the model's reliability, enabling us to confirm that biologically meaningful features drive AI predictions. Furthermore, by constructing valence‐specific embeddings via a Siamese network, we identified stable prototypes capturing shared facial configurations across animals, consistent with dimensional emotion models in humans.^[^ ^76^ ^]^ These prototypes robustly tracked emotional shifts, including pharmacologically induced changes, demonstrating their potential for generalizable emotion decoding across individuals and conditions. In addition to capturing pharmacologically induced emotional shifts, this framework also lays the groundwork for future studies examining how hallucinogens modulate the neural and behavioral correlates of emotion, offering potential insights into their therapeutic applications in affective disorders such as depression.

### 3.2 Characterizing Mice‐Specific Facial Expressions in Response to Emotional Changes

Our study reveals that certain facial actions can vary significantly even with the same emotional valence, underscoring the need for precise quantification to capture these dynamic expressions.^[^ ^17^, ^76^ ^]^ To address this complexity, we employed comprehensive multiparameter analyses using DeepLabCut‐based keypoint tracking, allowing fine‐grained, continuous quantification of facial features and visualizing distinct valence‐specific patterns. This extends foundational work by Dolensek et al. (2020),^[^ ^26^ ^]^ who demonstrated that mouse facial expressions reflect affective states using supervised CNN classification, but relied on discrete‐frame analysis and manual region labeling. By contrast, our approach enables continuous temporal tracking with higher spatial resolution, capturing dimensional valence dynamics more sensitively.

Among the various facial features, ear movements consistently emerged as particularly informative markers distinguishing positive and negative emotional states, aligning with prior findings from Lecorps and Féron (2015).^[^ ^79^, ^80^ ^]^ While human emotional expressions primarily involve the eyes and mouth,^[^ ^76^ ^]^ mice rely heavily on ear movements for both emotional signaling and environmental sensing,^[^ ^17^, ^79^ ^]^ reflecting evolutionary adaptations to their quadrupedal ecology and social context.^[^ ^81^ ^]^ Their highly mobile ears facilitate both ultrasonic communication and emotional expression,^[^ ^82^ ^]^ in contrast to human facial musculature optimized for complex social interaction.^[^ ^81^ ^]^

Previous studies have offered valuable observations on geometrical facial changes across emotional states, although certain methodological aspects have constrained the continuity and granularity of facial dynamics analyses. For example, Tanaka et al. (2023)^[^ ^25^ ^]^ and Moëne and Larsson (2023)^[^ ^80^ ^]^ used manual annotation on selected video frames, which limited continuous tracking. Syeda et al. (2024)^[^ ^28^ ^]^ proposed an automated framework based on 13 facial keypoints, but their model excluded ear‐related features and employed fewer spatial landmarks. Our approach combines high‐resolution spatial coverage with frame‐by‐frame tracking of geometrical parameters across multiple facial regions—including ear angle and shape—enabling a more temporally continuous and structurally refined analysis of facial dynamics. This comprehensive strategy allows for a more robust identification of the emotional significance of ear movements, providing stronger evidence for their role as reliable markers of affective valence in mice.

Importantly, the involvement of ear movements in emotional expression extends beyond rodents. Similar patterns have been observed in pigs during aggressive or evasive behaviors,^[^ ^83^ ^]^ and in horses and sheep, where ear positioning and movements have been shown to convey negative and positive emotional states.^[^ ^84^, ^85^ ^]^ The parallels in ear movements across species suggest shared structural and functional characteristics, indicating that various animals may use analogous biological strategies for emotional expression. Given that ear movements are relatively easier to capture in freely moving mice compared to other facial features, this offers a valuable opportunity to extend applications to naturalistic emotional monitoring. Collectively, our findings shed light on the evolutionarily conserved role of ear movements in affective communication and highlight their potential applications in animal welfare and translational emotion research across species.

### 3.3 Neural Correlates of Emotional Processing and the Extension of Valence Modulation across Broader Domains

To deepen our understanding of emotional processing in mice, it is essential to identify neural signatures that reflect these processes. In this study, we examined the relationship between facial expressions and neural activity in the VTA, a region involved in learning, addiction, and emotional regulation.^[^ ^36^, ^75^ ^]^ Prior research has shown that circuits controlling arousal and affective behaviors can be functionally dissociable.^[^ ^11^ ^]^ Consistent with these findings, our data demonstrate that valence‐specific facial expressions are tightly linked to dopaminergic and GABAergic activity within the VTA, while glutamatergic neurons primarily respond to high‐arousal stimuli independent of valence.^[^ ^72^, ^74^, ^86^ ^]^ This functional differentiation may reflect distinct upstream inputs: VTA glutamatergic neurons primarily receive inputs from cortical regions, dopaminergic neurons proportionally from the basal ganglia, and GABAergic neurons from the LHb and lateral dorsal tegmental nucleus.^[^ ^86^ ^]^

Supporting earlier studies, VTA glutamatergic neurons have been shown to respond to both positive and negative stimuli, playing a role in promoting wakefulness.^[^ ^74^ ^]^ Midbrain dopamine neurons are involved in reward processing, while VTA GABAergic neurons contribute to aversive responses and inhibit reward consumption.^[^ ^11^, ^72^, ^73^ ^]^ Although bitterness evoked a modest increase in VTA^DA^ activity, this likely reflects salience or fluid‐related motivation in water‐restricted animals, rather than positive valence. Such transient dopaminergic responses to salient but aversive stimuli have been reported previously.^[^ ^87^ ^]^ In our study, bitterness elicited weaker DA activity than water or sucrose but was accompanied by robust GABAergic activation and aversive facial expressions, supporting its classification as a negative stimulus.

Building on these observations, we further explored causal relationships between VTA activity and facial expressions using optogenetic manipulations. Consistent with prior reports that inhibiting VTA^GABA^ neurons reduces aversive flight responses,^[^ ^73^ ^]^ we observed that VTA^GABA^ inhibition altered negative facial expressions, particularly ear movements. Similarly, optogenetic inactivation of VTA^DA^ neurons affected positive facial expressions, specifically flattening ear dynamics, consistent with their established role in reward processing.^[^ ^88^ ^]^ Unlike Berridge and Kringelbach's prior work, which linked dopamine neuron activity to hedonic orofacial behaviors such as tongue protrusions and paw licks,^[^ ^8^, ^89^ ^]^ our approach highlights that ear movements are sensitive indicators of valence modulation. These findings show that VTA neuron‐type‐specific activity differentially shapes valence‐specific facial configurations, with ear movements acting as highly sensitive readouts of neural modulation.

While the VTA acts as a key integrator, emotional processing likely arises from distributed interactions across multiple brain regions. Parallel circuits in the amygdala and pontine central grey likewise encode positive and negative valence through distinct cell populations.^[^ ^10^, ^11^, ^90^ ^]^ Coordinated activity across these regions may converge onto shared facial motor pathways to generate nuanced emotional expressions. Notably, the extensive connections of the VTA, linking it to the hypothalamus, basal forebrain, insula, temporal lobe, NAc, and PFC, suggests a topological organization that supports its central role in coordinating emotional regulation.^[^ ^91^ ^]^ This anatomical convergence may offer valuable insights for future targeted neuromodulatory interventions, including deep brain stimulation for affective disorders.^[^ ^73^ ^]^

Our findings also suggest translational potential for real‐time emotional state decoding based on facial dynamics coupled to neural signatures. The tight correspondence between facial expressions and neuron‐type‐specific VTA activity supports the feasibility of closed‐loop brain–machine interface systems that monitor spontaneous affective fluctuations and deliver adaptive neuromodulation. Such frameworks align with emerging closed‐loop paradigms for internal state regulation in domains like pain control, where real‐time neural decoding guides targeted interventions.^[^ ^92^ ^]^ Although demonstrated here in head‐fixed mice, the principle of linking facial kinematics to underlying affective circuits may ultimately inform adaptive emotion‐modulating strategies across species and experimental contexts.

### 3.4 Limitations and Future Directions in Facial Expression Analysis

A primary limitation of our study is the use of head‐fixed mice in a controlled environment, which constrains natural variability in emotional responses and may limit ecological validity. Future research should adopt facial tracking systems for freely moving mice, allowing spontaneous and naturalistic expressions to emerge. Approaches such as head‐mounted cameras, multiangle recording platforms, or reflective pinna markers may enable robust ear‐focused tracking even under complex behavioral conditions.^[^ ^93^, ^94^, ^95^, ^96^ ^]^ Given the strong emotional sensitivity of ear dynamics observed in this study, such ear‐centric measures may serve as practical readouts for emotional state monitoring, especially in settings without specialized machine vision infrastructure. More flexible pose estimation frameworks, including SLEAP or ADPT, could further enhance motion tracking accuracy under challenging conditions such as rapid locomotion, occlusions, or multianimal interactions.^[^ ^97^, ^98^ ^]^ These tools would also facilitate 3D reconstruction of facial kinematics, yielding richer datasets for understanding dynamic emotional expressions.^[^ ^99^ ^]^

In addition to behavioral markers, integrating bioelectrical signals from central and peripheral systems may enrich emotional state assessment. Physiological measures such as electrocardiography and electromyography could provide complementary indices of arousal and motor activity, offering a multimodal framework for emotion decoding.^[^ ^100^ ^]^ Although technical constraints prevented their inclusion here, future incorporation of such signals could improve accuracy and translational relevance, particularly for cross‐species comparisons.

Environmental factors also warrant consideration. Captive laboratory mice often experience reduced sensory stimulation compared to wild counterparts, potentially altering emotional reactivity and generalizability.^[^ ^101^ ^]^ To improve ecological relevance, standardized housing protocols incorporating environmental enrichment, larger cages, and more naturalistic behavioral paradigms should be adopted. Furthermore, future studies should examine long‐term emotional dynamics, extending beyond short‐term fluctuations to capture sustained mood states and their influence on facial expression regulation. Including diverse mouse strains will also help establish the generalizability and robustness of facial‐expression‐based emotion decoding across genetic backgrounds.^[^ ^102^ ^]^

In summary, this study identified distinct facial expression patterns and associated neural signatures reflecting positive and negative emotional states in mice. Our AI‐based framework extracted interpretable geometric facial features underlying emotional processes that traditional statistical methods may overlook, revealing strong correlations between facial kinematics and VTA neuron‐type‐specific activity. These findings deepen our understanding of the neural substrates of emotion and lay the groundwork for translational strategies in emotion regulation. Ultimately, this work bridges animal models and human applications, offering a potential avenue for affective disorders through homology‐based emotional indicators.

## 4 Experimental Section

### Animal Preparation and Surgery

Adult (8–12 weeks) male C57BL/6J mice (Shanghai Bikai Keyi Biotechnology Co., Ltd., Shanghai, China), DAT‐Cre mice (courtesy of J. Hu, ShanghaiTech University), and Vgat‐Cre mice (courtesy of C. Li, National Laboratory of Lingang, China) were used. Animals were housed under a 12/12 h light/dark cycle, with a temperature of 22–25 °C and relative humidity of 65 ± 5%, with ad libitum access to food and water. All procedures were performed in accordance with guidelines approved by the Animal Ethics Committee of the Department of Laboratory Animal Science, Fudan University (Approval Nos. 202011019S and 202207015S).

A week prior to head‐fixed experiments, mice were anesthetized with isoflurane gas (induction: 4%; maintenance: 1–1.5%) for headplate surgery. After mounting the mouse in a stereotaxic apparatus (RWD Instruments), the scalp was sterilized, and a midline incision exposed the skull. Following skull cleaning with saline, a titanium headplate (1.1 g) was affixed with tissue adhesive and reinforced with dental cement, leaving an inner ring exposed for viral injections and optical fiber implants as necessary. For fiber photometry and optogenetics, a craniotomy was drilled over the target coordinates (VTA: AP −3 mm, ML ±0.98 mm, DV −4.24 mm, 8° angle), followed by the slow injection of indicated viruses (each 200–300 nL) using a Nanoject III (20 nL min^−1^). Postinjection, the pipette was held in place for 15 min before fiber implantation, securing fibers with dental cement. Mice not undergoing optogenetic or fiber procedures had the headplate fully covered. Postsurgery, animals were water‐restricted and given ≈1.5 g of hydrogel daily to maintain 85–90% of their initial body weight. During recovery (minimum seven days), metal food trays were removed, and food was placed directly on the cage floor to prevent dislodging the implanted headplate.

After the headplate surgery, an intraoral cheek fistula could be implanted, following a previously described procedure.^[^ ^48^ ^]^ An incision was made in the cheek near the first molar, and another was made in the scalp. A 30 mm Silastic tubing (0.30 mm I.D., 0.46 mm O.D.) was inserted through the cheek incision into the oral cavity and tunneled subcutaneously to the scalp. At the scalp incision, the Silastic tubing was connected to an L‐shaped stainless steel tube. A piece of polyethylene tubing was fitted to the exposed end of the stainless steel tube and sealed with a plug to prevent blockage.

### Behavioral Experiments

To reduce the stress levels of mice during experiments, it was essential to first habituate them to head fixation and voluntary locomotion on the treadmill. This habituation process began by gradually increasing the duration of head fixation over 3–6 days, depending on the individual mouse's response. During this period, mice were trained to drink water by licking from a cannula, ensuring they learned to obtain water voluntarily during head fixation. Habituation sessions began with 15 min on the first day, increasing to 30 min on the second day, and extending to 60 min from the third day onward. Additionally, in each session where head fixation was reapplied, the mice were allowed at least 30 min to recover and reacclimate before starting the experiment.

### Stimulus Timeline

In this study, excluding drug treatments, each stimulus was presented for 5 s to maintain data consistency across trials. Different categories of stimuli were presented in a pseudorandomized order. During fiber photometry and optogenetics, the stimulus duration was shortened to 2 s to synchronize with neural recording, with a fixed stimulus order. Identical stimuli were applied across 3–5 trials; if the mouse failed to engage in licking behavior, fewer trials were performed to avoid interference with subsequent tasks. A 5 min interval was maintained between individual trials, and a 15 min interval was set between different stimuli to prevent habituation or carryover effects.

### Tastant Administration Protocol

In this study, four different lick‐based stimuli were applied to the mice: two positive tastants (4% sucrose and 75 mm NaCl, both obtained from Sinopharm Group Chemical Reagent Co., Ltd., Shanghai, China), one aversive stimulus (5 mm denatonium benzoate, representing bitterness, obtained from Juxing Biological Technology Co., Ltd., Henan, China), and regular drinking water serving as a neutral control. These tastants were delivered to the mouse's mouth through a plastic tube connected to a peristaltic pump. To prevent cross‐contamination between different solutions, the system was flushed with drinking water for 3 min after each type of tastant.

### Electrical Shock Application

In this study, 0.4 mA electrical shocks were administered to the tail of each mouse using a VanBi precision shocker. A small cylindrical plastic holder was threaded through the tail to secure it at the midsection. This setup allowed for the accurate positioning of two copper electrode pads on opposite sides of the tail, ensuring continuous and consistent stimulation during the 2–5 s shock period.

### Drug Administration

In the first phase of the experiment, mice were head‐fixed and left undisturbed for 15 min to establish a baseline. Afterward, the animals were intraperitoneally (i.p.) injected with one of the following solutions: lithium chloride (0.15 m in 0.9% saline, administered at 20 mL kg^−1^ body weight, obtained from Sinopharm Group Chemical Reagent Co., Ltd., Shanghai, China), ethanol (12.6% v/v in 0.9% saline, administered at 1.5 g kg^−1^ body weight, obtained from Sinopharm Group Chemical Reagent Co., Ltd., Shanghai, China), or DOM hydrochloride (1 mg kg^−1^ in 0.9% saline, administered at 10 mL kg^−1^ body weight; DOM hydrochloride was provided by Dr. Yan Haitao, State Key Laboratory of National Security Specially Needed Medicines, Beijing, China). In all groups, control animals were injected with an equivalent volume of 0.9% saline. Observations resumed 1 min after injection and continued for 60 min.

### Facial Videography and Processing

In this study, videography was conducted under dark conditions, using a Jet Ball system (PhenoSys GmbH) that allowed head‐fixed mice to simulate free movement on an air‐cushioned spherical treadmill. The treadmill consisted of a 20 cm polystyrene ball supported by a pressurized airflow system, enabling near‐effortless movement for the mice. High‐resolution facial expressions were recorded using an infrared camera (DALSA, G3‐GM11‐M2020) equipped with a 16 mm prime lens and an 850 nm infrared filter (30 nm cutoff). The camera operated at 30 frames s^−1^, combined with a 15 W 850 nm infrared light source (IR120‐IR‐24), to capture facial expressions without introducing sensory interference (Figure 1A). The camera was positioned at a 90° angle to the mouse to ensure optimal facial recording. The entire experimental session, including adaptation periods, prestimulus intervals, and interstimulus phases, was continuously recorded for subsequent analysis (Figure S1A, Supporting Information).

Facial expression changes were analyzed by focusing on the 1 min intervals before and after the application of sensory stimuli such as sucrose, NaCl, bitterness, and shock. Drug treatments were evaluated by analyzing facial expressions for 15 min prior to, and 60 min following, intraperitoneal injections. For video processing, RGB‐frames were extracted from AVI video files using Python's OpenCV library,^[^ ^103^ ^]^ while HOG features (8 orientation bins, 16 × 16 pixels per cell, 1 × 1 cells per block, input size 1024 × 1024, grayscale) were computed using the scikit‐image library to represent the shape and appearance of mice's facial expressions.^[^ ^104^ ^]^

### Fiber Photometry Recording and Processing

For fiber photometry experiments, AAV‐EF1α‐DIO‐GCaMP6f‐WPRE‐hGH polyA (AAV2/9, 5.0  ×  10^12^ vg mL^−1^, from BrainVTA) was injected into the VTA of DAT‐Cre or Vgat‐Cre mice to detect calcium concentration changes in DA and GABAergic neurons. Meanwhile, AAV‐CaMKIIa‐GCaMP6f‐WPRE‐hGH polyA (AAV2, 5.0  ×  10^12^ vg mL^−1^, from BrainVTA) or AAV‐hSyn‐GCaMP6f‐WPRE‐hGH polyA (AAV2, 5.0  ×  10^12^ vg mL^−1^, from BrainVTA) was injected into the VTA of C57BL/6J mice to monitor calcium dynamics in glutamatergic neurons and pan‐neuronal populations, respectively. An optical fiber photometry system (410/470 nm, Inper) was used to record real‐time calcium signals in freely behaving mice. The system utilized 410 and 470 nm dual LED light sources, with each wavelength reflected by a dichroic mirror and coupled through the optical fiber via an eyepiece lens (Figure 6A). The system used bundled fiber optic cables (Inper) with a 200 µm core diameter and numerical aperture (NA) 0.37. Excitation light power was set to ≈30 µW for the 470 nm LEDs, and ≈15 µW for the 410 nm LED. The 470 nm LED light triggered calcium‐dependent fluorescence from GCaMP6f, corresponding to neuronal activity, while the 410 nm LED light excited calcium‐independent fluorescence, serving as a control isosbestic channel to account for noise and movement artifacts. The emitted fluorescence signals were focused on a CMOS camera sensor for detection. The total sampling rate was set at 30 Hz, with each light source sampling at 15 Hz to ensure accurate time‐resolved measurements of neuronal activity. Additionally, during each emotional stimulus, external devices sent TTL signals to synchronize and mark the onset of stimuli within the data, ensuring precise temporal correlation between neuronal activity and behavioral events.

For signal processing and analysis, fluorescence signals were corrected for photobleaching using the adaptive iteratively reweighted penalized least squares method,^[^ ^105^ ^]^ effectively eliminating gradual signal decay over time. Following this, motion correction was performed by fitting the 410 nm control signal to the 470 nm calcium signal using least squares linear regression. The resulting 410 nm component was subtracted from the 470 nm signal to yield motion‐corrected calcium fluorescence data. Calcium transients (Δ*F*/*F*) were then calculated using the formula: Δ*F*/*F* = (signal − fitted control)/fitted control, with the fitted control representing the motion‐corrected signal. To analyze responses to sensory stimuli, peristimulus time histograms (PSTHs) were generated.^[^ ^106^ ^]^ The baseline‐corrected fluorescence intensity (Δ*F*/*F*) for each trial was calculated by subtracting the average of the 4 s prestimulus baseline from the raw Δ*F*/*F* signal of the trial. These baseline‐corrected trials were averaged to produce the final PSTH traces. Calcium responses to each stimulus were standardized by calculating the ratio of each stimulus's peak calcium transient to the neutral water response's peak calcium transient, thereby emphasizing each stimulus's impact relative to a neutral baseline and highlighting stimulus‐specific differences (Figure 6C–F). Additionally, *z*‐scoring was applied across subjects and sessions to support correlation analyses between facial expressions and neuronal activity (Figure 7A,B). Peak detection, correlation analysis, and heatmap visualizations were performed using custom‐written MATLAB scripts.

### Optogenetic Manipulation In Vivo

For optogenetic manipulations, 200 nL of AAV‐hEF1a‐DIO‐eNpHR3.0‐EYFP‐WPRE‐Pa (AAV2/5, 5.0  ×  10^12^ vg mL^−1^, from Taitool Bioscience) or control virus (AAV2/9, 5.0  ×  10^12^ vg mL^−1^, from Taitool Bioscience) was injected bilaterally into the VTA of DAT‐Cre or Vgat‐Cre mice (Figure 7C,H). To stimulate eNpHR‐expressing DA or GABA axons in the VTA, a yellow laser light (589 nm) controlled by an intelligent optogenetics system (IOS‐589, RWD) was applied through an implanted optical fiber. The fibers used for light delivery had a core diameter of 200 µm and a NA of 0.37, and were assembled with 1.25 mm ceramic ferrules (Inper). The light power at the fiber tip was set to 10 mW. A train of pulses was delivered at 20 Hz with a pulse width of 10 ms. Sensory stimuli were synchronized with the optogenetic inhibition: the light stimulation lasted for 5 s, while the sensory stimulation lasted for 2 s. This setup ensured precise temporal alignment between optogenetic and sensory stimuli, allowing for the assessment of VTA neuron inhibition on emotional processing.

### Histology and Microscopy

Mice were deeply anesthetized with 0.01 mL g^−1^ of 4% chloral hydrate administered via intraperitoneal injection. After confirming anesthesia through the absence of reflexes, mice were perfused transcardially with 1× phosphate‐buffered saline (PBS), followed by ice‐cold 4% paraformaldehyde (PFA). Postperfusion, the brain was extracted and fixed in 4% PFA at 4 °C overnight. Dehydration was carried out in 20% sucrose for one day, followed by 30% sucrose for an additional day. Coronal brain sections (30 µm) were sliced using a cryostat (Leica) and identified based on anatomical markers relevant to the VTA.

For immunohistochemistry, the sections were washed 3 times in PBS and permeabilized with 0.3% Triton X‐100 for 30 min at room temperature. After blocking for 1 h with a solution containing donkey serum, sections were incubated overnight at 4 °C with the following primary antibodies: mouse anti‐TH (AMAB91112, Sigma‐Aldrich), mouse anti‐CaMKII (ab22609, Abcam), and rabbit anti‐GAD65/67 (G5163, Sigma‐Aldrich). The next day, sections were washed in PBS and incubated with secondary antibodies (Alexa Fluor 647 Donkey anti‐Mouse IgG (H+L), A31571, Invitrogen or Alexa Fluor 647 Donkey anti‐Rabbit IgG (H+L), A31573, Invitrogen) for 1 h, followed by nuclear staining with DAPI. Finally, the sections were washed 3 times in PBS, mounted on slides, and sealed with coverslips using a mounting medium. Fluorescence images were captured using a confocal microscope (Olympus), and the signals were analyzed with ImageJ to confirm viral infection and trace neuron populations.

### Facial Expression Analysis—Motion Energy of Mouse Face

Motion energy^[^ ^107^ ^]^ was calculated by measuring the difference between consecutive frames for each pixel within the specified facial region. To capture distinct facial movements, side‐view recordings were processed to generate heatmaps that highlighted regions of facial activity through mean pixel‐based changes in motion energy during stimulus presentations. This visualized approach allowed to observe the activation of specific facial regions in response to various stimuli, facilitating precise visualization of region‐specific activity patterns associated with each emotional response (Figure 1B and Figure S5A (Supporting Information)).

Average motion energy was calculated by summing pixel‐wise frame‐to‐frame differences, then normalizing by the total number of pixels for each condition: saline, ethanol, LiCl, and DOM (Figure S3B, Supporting Information).

### Network Training

The AI‐based model, utilizing ResNet18 architecture with weights pretrained on ImageNet, was designed to classify facial expressions of mice in response to diverse stimuli (Figure 1D). The dataset included 10 mice, each contributing up to five trials per stimulus type. For each mouse, 3–4 trials per stimulus were used for training and validation (random 80/20 split), while one additional trial per stimulus was reserved for testing in nine animals (one excluded due to insufficient usable trials). To ensure independent evaluation, no test trials were included in the training or validation sets. The model was trained and evaluated on a frame‐by‐frame basis using individual frames extracted from the 0–2 s window following stimulus onset, to focus on the period of maximal stimulus‐evoked facial expressions. Data augmentation^[^ ^108^ ^]^ techniques were applied to enhance model robustness, including random resizing, ±10° rotation, and normalization according to standard image statistics. Images were resized to 224 × 224 pixels to meet ResNet18 input requirements. The model was trained over 50 epochs with a batch size of 256, using stochastic gradient descent with momentum, a learning rate of 0.001, and the L2 norm of the weights, with cross‐entropy loss guiding optimization. Performance evaluation included accuracy metrics and confusion matrix analysis. All model training and evaluations were conducted in Python with PyTorch (version 1.3.1) on a CentOS 7.9 system, utilizing PyCharm as the integrated development environment.^[^ ^109^ ^]^ The computational setup included dual Intel Xeon Gold 5120 CPUs (2.2 GHz, 14 cores each) and three NVIDIA 1080 GPUs, providing extensive computational resources to support the demands of training and evaluation.

### Siamese Network Architecture

This approach employed a Siamese Network with two identical ResNet18‐based subnetworks, each serving as a feature extractor with shared weights (Figure 2A). Each image pair—composed of either “same‐valence” or “different‐valence” expressions—was input into the two sub‐networks, allowing the model to learn consistent feature representations across both branches. This architecture enabled the network to simultaneously learn both feature extraction and similarity measurement, rather than relying on a predefined similarity metric. This design encouraged the learning of generalizable, valence‐specific features that were independent of individual identity. Feature vectors from each image were subsequently compared by calculating the Euclidean distance,^[^ ^110^ ^]^ which quantified the similarity between expressions. The embedding space was refined using a contrastive loss function that reduced distances between “same‐valence” pairs while increasing separation for “different‐valence” pairs.^[^ ^111^ ^]^ A margin parameter, set based on the average squared pairwise distance, reinforced this separation and improved generalization across varied facial expressions. Training pairs were constructed from 13 mice, and the model was evaluated on five independent mice not included in training, ensuring across‐subject generalization. Specifically, for paired output vectors output_1_ and output_2_, the contrastive loss function was defined as

(1) $$L=\frac{1}{2N}\sum_{i=1}^{N}\left[y_{i}\cdot d_{i}^{2}+\left(1-y_{i}\right)\cdot {\left\{\max\left(m-d_{i},0\right)\right\}}^{2}\right]$$

where *N* is the number of training pairs, *y_i_* is the label (1 for similar pairs, 0 for dissimilar), *d_i_* represents the Euclidean distance between the feature embeddings output_1_ and output_2_ for each image pair, calculated as *d_i_* =  ∥output_1_ −  output_2_∥, and *m* is the margin, encouraging embeddings of similar pairs to stay close and dissimilar pairs apart. The margin was empirically set based on the average interpair distance in the training set.

### Grad‐CAM for Facial Feature Visualization

To analyze the features contributing to classifications of emotional expressions within the end‐to‐end deep learning network, Grad‐CAM was applied: a technique used to assess which image regions hold the most relevance in the class predictions of the network. By calculating importance scores for each spatial position (*x*, *y*) within the last convolutional layer, this method assigned visual significance across the input image regions. These importance scores resulted from a linear combination of the activations, weighted by their respective output weights for the predicted class. The resulting class activation map was then resampled to match the size of the original input image, providing an overlay that highlighted critical regions (Figure 4A).

In the resulting heatmaps, red areas highlighted regions with high discriminative importance for the target class, while blue areas indicated low‐relevance regions. Through this approach, key facial features were consistently identified, such as the oral region, whiskers, snout, and ears, as the primary drivers in the classification of emotional expressions. These heatmaps enabled to confirm that the model captured generalized, class‐relevant features rather than overfitting to individual‐specific or isolated regions, ensuring its robustness across diverse emotional states and stimuli.

To identify which facial regions contribute to similarity assessments in the Siamese network, an adaptation of Grad‐CAM tailored was implemented for similarity‐based models.^[^ ^53^, ^54^, ^55^ ^]^ Each image in a pair was passed through the same convolutional backbone to extract fixed‐length embeddings. The Euclidean distance was then computed between embeddings as the similarity metric. Following recent frameworks for explainable image similarity, Grad‐CAM was applied to the final convolutional layer of the backbone network in each branch. Specifically, the Euclidean distance was used as the scalar target for backpropagation. For same‐valence image pairs, the negative distance was used to emphasize features contributing to similarity; for different‐valence pairs, the positive distance highlighted features driving dissimilarity. This strategy allowed to generate class‐independent saliency maps that revealed the facial regions most influential in the network's similarity judgments. All saliency maps were normalized and overlaid onto the original images for visualization (Figure S2C, Supporting Information).

### Dimensionality Reduction and Clustering

The high‐dimensional HOG features extracted from facial images were reduced to 2D to visualize the distribution of facial expressions across various emotional stimuli in an unsupervised manner. Dimensionality reduction was first conducted with PCA, reducing the data, originally at a resolution of 1024 × 1024 pixels, to 400 dimensions, followed by UMAP to create a 2D embedding. *k*‐means clustering was then applied to identify distinct expression groups.^[^ ^112^ ^]^ All analyses were performed in Python 3.7.0.

PCA reduced data complexity by extracting principal components that retained essential variance from the original features. UMAP subsequently mapped these components onto a low‐dimensional manifold, preserving local neighborhood relationships and capturing complex, nonlinear patterns within the data. Parameter tuning led to setting *n*_neighbors in UMAP to 45 to optimize the balance between local and global structure, ensuring that similar data points from the high‐dimensional space remained in close proximity within the 2D representation. Following dimensionality reduction, *k*‐means clustering was used to group the expressions (Figure 1G and Figure S1D (Supporting Information)). This clustering enabled a deeper analysis of facial expression distributions, highlighting differences and commonalities in facial features across the emotion categories.

### Coregistration of Facial Images across Mice

To achieve cross‐subject alignment of facial frames, frames from the neutral phase of each session were selected, and six key facial landmarks were manually identified: the nose tip, inner and outer eye corners, the anterior edge of the lower lip, cheek, and the posterior edge of the pinna (Figure 2E and Figure S3A (Supporting Information)). These landmarks served as reference anchors for alignment to a standardized prototype image. An affine transformation was calculated from these points, providing an initial alignment further optimized through translation, rotation, and scaling. This refinement minimized Euclidean distances between the corresponding landmarks and contour similarity across key regions, yielding consistent facial alignment across sessions and subjects. Derived transformation parameters were applied across each frame in the session, ensuring synchronized feature positioning for multiple mice and sessions. To evaluate alignment quality, color‐coded overlays were created by showing unaligned images in the red channel and aligned images in green and blue channels (Figure S3A, Supporting Information). The degree of channel overlap offered a direct visual measure of alignment accuracy across sessions. This alignment procedure was only applied to analyses requiring cross‐subject similarity comparisons to prototypical facial expressions (cosine similarity to positive or negative prototypes), and was not used in motion energy computation or automated classification analyses.

### Valence‐Specific Prototype Extraction Method

To generate valence‐specific prototypes that effectively capture representative facial expressions for different emotional valences in mice,^[^ ^78^ ^]^ a two‐stage selection process was implemented (Figure 2C,D). First, subprototypes were identified by selecting frames that maximized dissimilarity from the neutral baseline while maintaining high similarity within the same stimulus category. Specifically, the cosine similarity was calculated between each frame's HOG feature vector during stimulus presentation and both the HOG vector of the neutral baseline as well as other HOG vectors within the same stimulus category.^[^ ^113^ ^]^ Frames that demonstrated minimal similarity to the neutral baseline and maximal similarity within the stimulus group were chosen to ensure that each selected frame was both distinct from the neutral expression and highly representative of its specific stimulus.

The cosine similarity between two vectors *a* and *b* was computed using the formula

(2) $$\cos\left(a,b\right)=\frac{a\cdot b}{∥a∥∥b∥}$$

Finally, to obtain a comprehensive valence‐specific prototype, the HOG representations of subprototypes within each valence category were averaged. This resulted in a unified representation encapsulating the most distinguishing features for positive stimuli (e.g., sucrose, NaCl) and negative stimuli (e.g., bitterness, tail shock). All the prototypes were generated using data from a specific mouse that was excluded from all other analytical processes. This mouse was selected prior to analysis based on good surgical recovery (e.g., no head swelling or wound artifacts), stable head‐fixation, and complete, high‐quality recordings across all stimulus conditions.

### DeepLabCut Tracking and Quantification of Facial Features

DeepLabCut (version 2.2.3) was utilized to track the facial movements of mice, focusing on key areas such as the nose, mouth, whiskers, ears, eyelid diameter, and pupil diameter, based on side‐view images. For each animal, video segments corresponding to stimulus periods were extracted, and 20 representative frames were randomly selected for manual labeling. A total of 37 facial keypoints were annotated per frame using DeepLabCut's GUI (Figure 4B). Additional frames were labeled iteratively if initial tracking performance was suboptimal. The model was trained using a ResNet‐101 backbone for 1 000 000 iterations with a batch size of 2. Input frame resolution was set to 2064 × 1544 pixels. After training, the model automatically tracked key points on unlabeled videos, generating pixel coordinates for each key point before and after different emotional stimuli. Keypoints with confidence scores below 0.9 were discarded or linearly interpolated. All coordinate trajectories were smoothed using a 5‐frame median filter to reduce jitter.

To provide a more detailed overview of the keypoint architecture, the spatial configuration of all 37 annotated landmarks across facial regions was described below. For the nose, key points were placed at the anterior tip and the top of the nose bridge, along with three points forming an inverted triangle around the left nostril. The mouth was positioned using three key points: one at the lower lip's anterior edge, one at the center as a reference point to reduce drift, and a third at the outer left mouth corner. Whisker tracking involved selecting three prominent whiskers visible across most camera angles, marking four key points from the base to the tip. Ear movements were tracked by placing a key point at the posterior edge of the pinna, representing the furthest ear movement, and two additional points along the pinna for width, plus a key point at the ear's root near the auditory canal. For eyelid tracking, four key points were labeled horizontally and vertically, representing the farthest displacements of the eyelid. Pupil diameter was tracked using eight points—four cardinal and four diagonal—allowing precise measurement of horizontal and vertical changes in pupil size.

Based on these spatial annotations, region‐specific features—such as distances, angles, and directional angles—were computed to quantify facial dynamics over time. The size of the pupil was determined by measuring the vertical distance between the top and bottom key points, where larger distances indicated dilation and smaller ones reflected constriction (Figure 4D). Eye opening was quantified by the distance between the upper and lower eyelid markers, with greater distances suggesting more open eyes (Figure S4A, Supporting Information). Mouth inclination was calculated from the angle formed between the nostril, inner eye corner, and the anterior edge of the lower lip, with larger angles corresponding to a more open mouth (Figure 4E). The snout–mouth length was defined as the distance between the anterior tip of the nose and the lower lip, where smaller distances suggested a retracted snout and increased facial contraction (Figure 4F). Nose position was represented by the distance between the anterior tip of the nose and the nose bridge, with greater distances indicating an extended nose posture (Figure S4B, Supporting Information). Whisker movement was measured by the angle formed between the base and tip of a selected whisker relative to the negative *x*‐axis, with smaller angles representing forward‐directed whiskers (Figure 4G). Ear length was defined as the distance between the posterior edge of the pinna and the ear base (Figure 4H), while ear movement was measured by the angle between the posterior edge of the pinna and the *x*‐axis, where larger angles indicated raised ears (Figure 4I). Finally, ear width was measured as the distance between mid‐ear points (Figure S4C, Supporting Information), and ear fold was determined by the angle between the ear base, the posterior edge of the pinna, and the mid‐ear point, with smaller angles indicating more folded ears (Figure S4D, Supporting Information).

For each stimulus, the change relative to the baseline was calculated by measuring how much the observed value during the stimulus deviated from the baseline, and this deviation was expressed as a proportion of the baseline value. The baseline itself was defined as the average value recorded during the 4 s window prior to the stimulus. This method was used in general analyses to quantify deviations from baseline on a standardized scale. In Figure 4, an additional normalization strategy was applied to improve the comparability of facial responses across different emotional stimuli. For each trial, the proportional change was first calculated relative to the prestimulus baseline, and then corrected by subtracting the corresponding proportional change observed in the temporally nearest water control trial. These proportional changes were expressed as percentages for visualization. Given the small magnitude of facial movements and the dynamic nature of emotional states, this approach ensured that the differences observed in Figure 4 were reflected stimulus‐specific emotional effects rather than nonspecific baseline fluctuations.

### LSTM‐Based Classification of Region‐Specific Facial Dynamics

To evaluate the contribution of dynamic facial features to emotion classification, a LSTM‐based classification model was implemented using DeepLabCut‐tracked keypoints. For each trial, the (*x*, *y*) coordinates of four selected keypoints from a specific facial region (ear, snout, whiskers, or pupil) were extracted over a 2.5 s window, spanning from 0.5 s before to 2 s after stimulus onset. This analysis was conducted using data from 5 mice with manually annotated facial keypoints. Within each region, the four keypoints with the highest variance across trials were selected to maximize discriminative potential.

The data were *z*‐scored and downsampled by retaining every third frame, resulting in 25 timepoints per trial. Input sequences were reshaped into tensors and fed into an LSTM classifier comprising three hidden layers with 256 units each and a dropout rate of 0.3. The model was trained to classify five stimulus conditions (sucrose, NaCl, bitterness, shock, and neutral) using tenfold stratified cross‐validation. To prevent information leakage, data were split at the trial level, ensuring that frames from the same trial did not appear in both training and test sets. Classification performance was evaluated by averaging decoding accuracy across folds.

### Occlusion Sensitivity Analysis with Tenfold Cross‐Validation

In this study, a modified occlusion analysis approach^[^ ^114^ ^]^ was implemented to assess both the significance of specific facial regions in classifying emotional expressions and the impact of model retraining on partially occluded datasets. Neutral‐colored masks were systematically applied to preidentified facial regions that prior experiments indicated as highly relevant to emotion changes (Figure 5F). This allowed to precisely measure each area's contribution to classification accuracy.

Unlike traditional occlusion methods, which typically kept model parameters fixed, the model with each masked dataset was retrained to compare feature extraction shifts resulting from occlusion. This analysis used data from 10 mice and was performed with tenfold cross‐validation (Figure 5G). To avoid data leakage, all frames from a given trial were kept within the same fold during splitting. In each fold, nine segments were used for training, while the remaining segment was held out for validation, yielding ten distinct performance metrics. Mask sizes were optimized to fully cover designated regions of interest, improving interpretability and enabling region‐specific insights. Reductions in classification accuracy, especially within regions sensitive to emotional change, underscored the critical role of these areas in accurate emotion detection. Notably, the accuracy obtained under the no‐mask condition in this analysis might be slightly lower than that reported in Figure 1F, as the 10‐fold cross‐validation framework imposed a stricter evaluation by requiring retraining from scratch for each fold and ensuring trial‐level separation between training and validation data.

### Statistics

All statistical analyses were conducted using Matlab and GraphPad. Statistical significance was determined with a threshold of *p* < 0.05. For multiple post‐hoc comparisons, the Dunn's test and Bonferroni correction were used to adjust for multiple comparisons.

## Conflict of Interest

The authors declare no conflict of interest.

## Author Contributions

Y.C. conceived and designed the study, conducted the main experiments, and drafted the paper with contributions from all authors. R.H. carried out virus injections, stereotaxic surgeries, and contributed to data analysis. Y.C. and Z.C. developed machine learning algorithms. Y.C., Z.C., and J.L. contributed to data collection and analysis. S.C. and S.X. assisted in interpreting results. J.F. and T.W.R. provided critical intellectual revisions. H.Y. supplied DOM and conducted pilot drug treatment experiments. X.X. supervised the study, validated findings, and contributed to paper review and editing. All authors reviewed and approved the final paper for submission.

## Supporting information



Supporting Information

Supplemental Movie 1

Supplemental Movie 2

Supplemental Movie 3

Supplemental Movie 4

## Data Availability

The data that support the findings of this study are available from the corresponding author upon reasonable request.
