# Supplementary material for: Cracking the Valence Code: Patterned Facial Kinematics and Neural Signatures of Emotional Expressions in Mice
Source: Adv Sci (Weinh). 2025 Aug 20;12(42):e17156. doi: 10.1002/advs.202417156 (PMC12622411; doi:10.1002/advs.202417156)
Supplement: Supplementary file 1 — Supporting Information [file ADVS-12-e17156-s003.pdf]

Supporting Information

Cracking the Valence Code: Patterned Facial Kinematics and Neural Signatures of Emotional Expressions in Mice

*Yujia Chen, Ruiqing Hou, Zhinan Chen, Junli Lu, Si Chen, Shisheng Xiong, Jianfeng Feng, Trevor W. Robbins, Haitao Yan, and Xiao Xiao**

Supplementary Figures

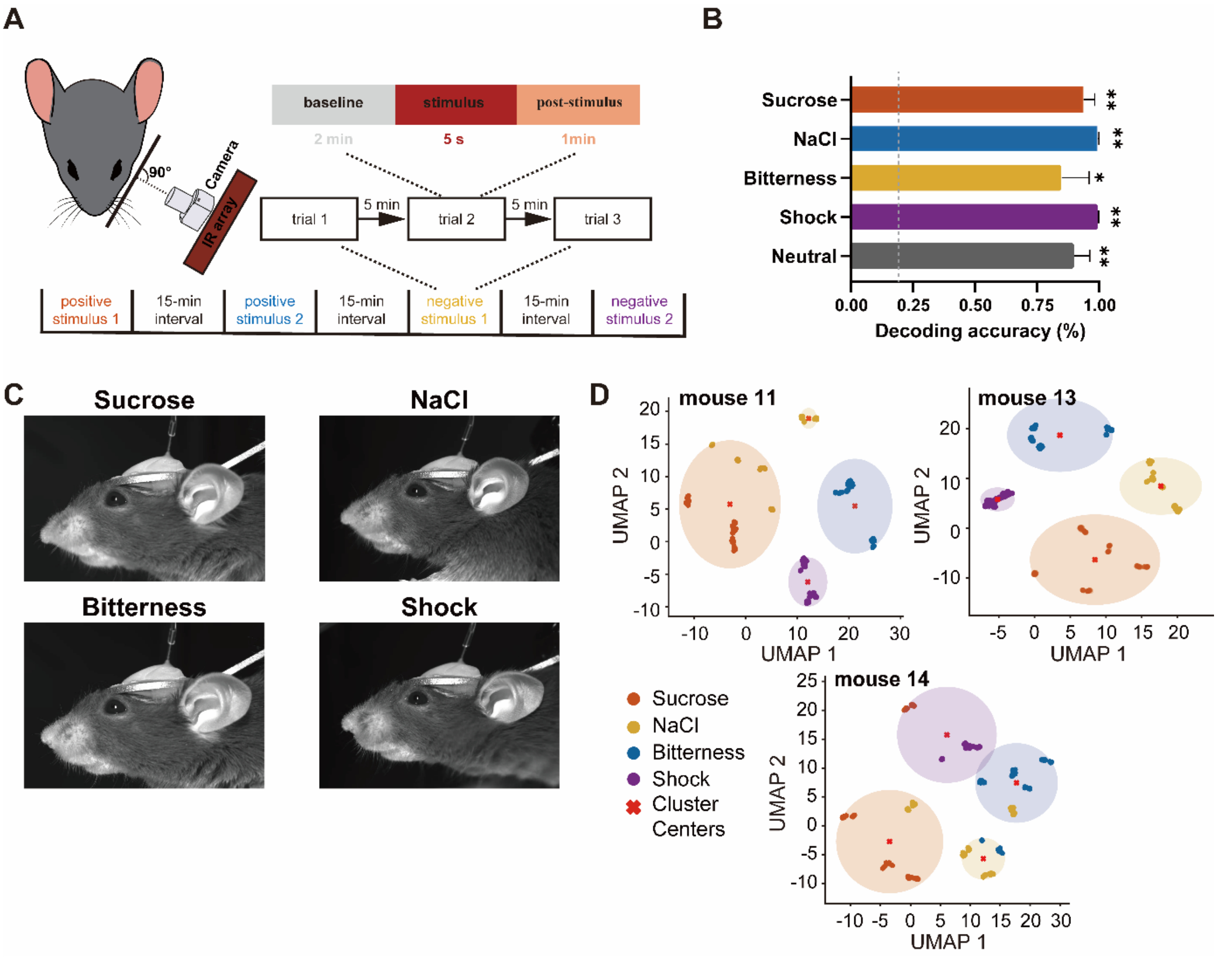

Figure S1. Stimuli-evoked facial expressions align well with the valence model. (A) Schematic for the arrangement of orofacial videography. Device setup: to effectively capture facial expressions, the camera was oriented perpendicular to the mouse's side profile. Stimulus timing and presentation: each of the 4 stimuli was applied for 5 seconds after a 2-minute baseline, followed by 1 minute of recording, with 3 repetitions spaced 5 minutes apart (unless the mouse did not engage in licking behavior during liquid presentation). A 15-minute interval was maintained between different stimuli. (B) Decoding accuracy for each facial expression in the test dataset (N = 9 mice) in response to different stimuli. *p < 0.05, **p < 0.01, one sample, two-sided Wilcoxon test with Bonferroni correction. (C) Facial responses in one exemplary mouse during the delivery of intraoral solutions (sucrose, NaCl, or bitterness) through a cheek fistula and during tail shock (Movie S1 and S2). (D) UMAP visualization of facial expression values from each cheek-fistula-implanted mouse in response to all stimuli. Red marks indicate k-means cluster centers; shaded circles represent the cluster boundaries defined by the maximum distance from their centers.

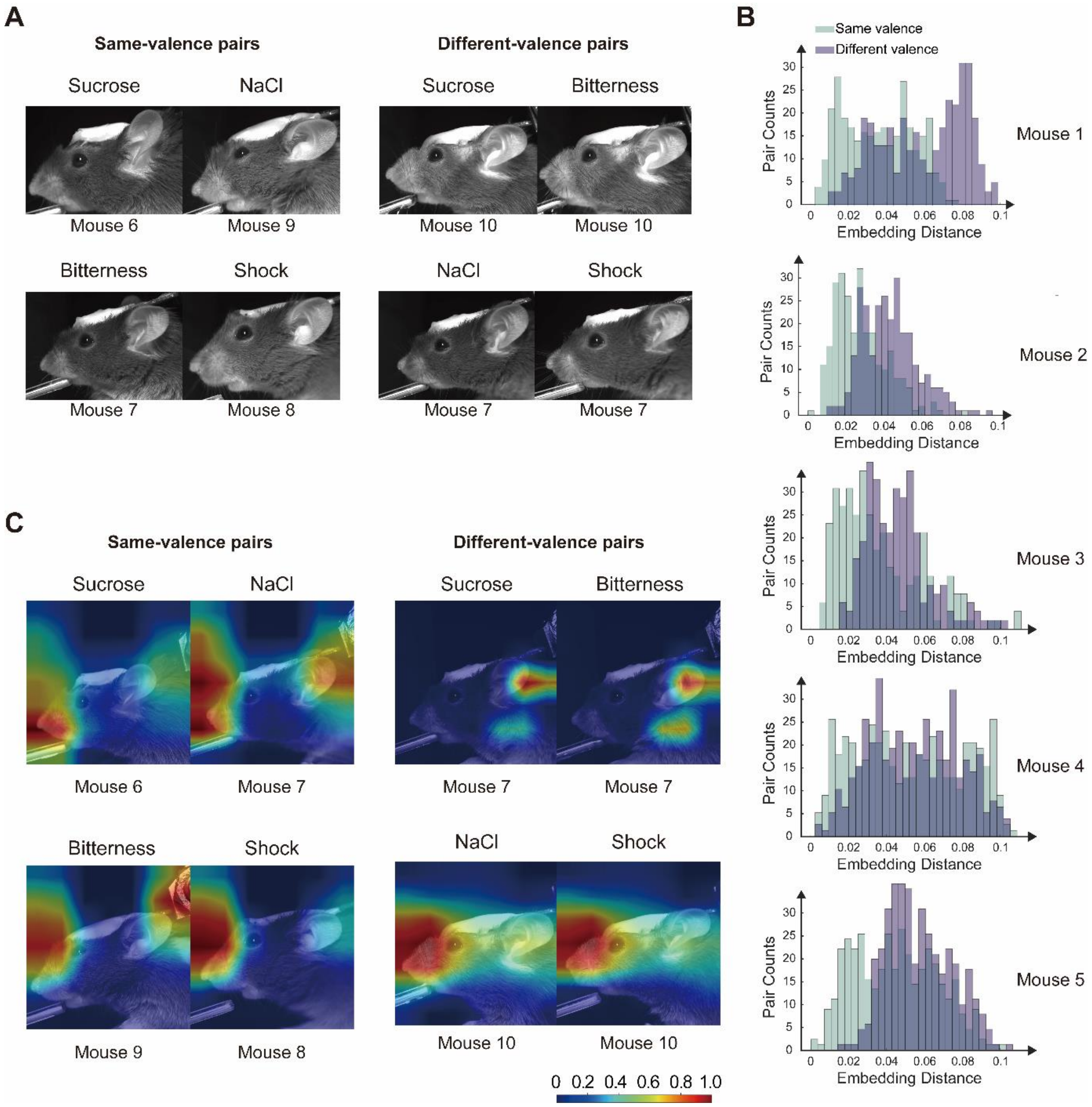

Figure S2. Revealing differences in mice's facial expressions under positive and negative valence using a Siamese network. (A) Example same-valence and different-valence pairs in training dataset. Same-valence pairs were not from the same animal. Same-valence pairs originate from different animals, whereas different-valence pairs come from the same animal. (B) Distributions of embedding distances for facial expressions in same-valence pairs shown in green and different-valence pairs shown in purple for each individual. Significant differences were observed in four out of five animals ($p < 0.001$, two-sided Welch's t-test); one mouse showed no significant difference ($p = 0.28$). These findings support the consistency of valence-specific embedding structures across individuals, complementing the group-level pattern shown in Figure 2B. (C) Adaptation of Grad-CAM for the Siamese network. For same-valence image pairs, the heatmaps highlight salient facial regions (ears, mouth, and snout) that consistently contribute to perceived similarity across animals (higher heatmap values indicate stronger influence on similarity judgments). For different-valence pairs, heatmaps reveal features contributing to dissimilarity, predominantly localized to the ears and whisker pads (lower heatmap values reflect greater influence on dissimilarity assessments).

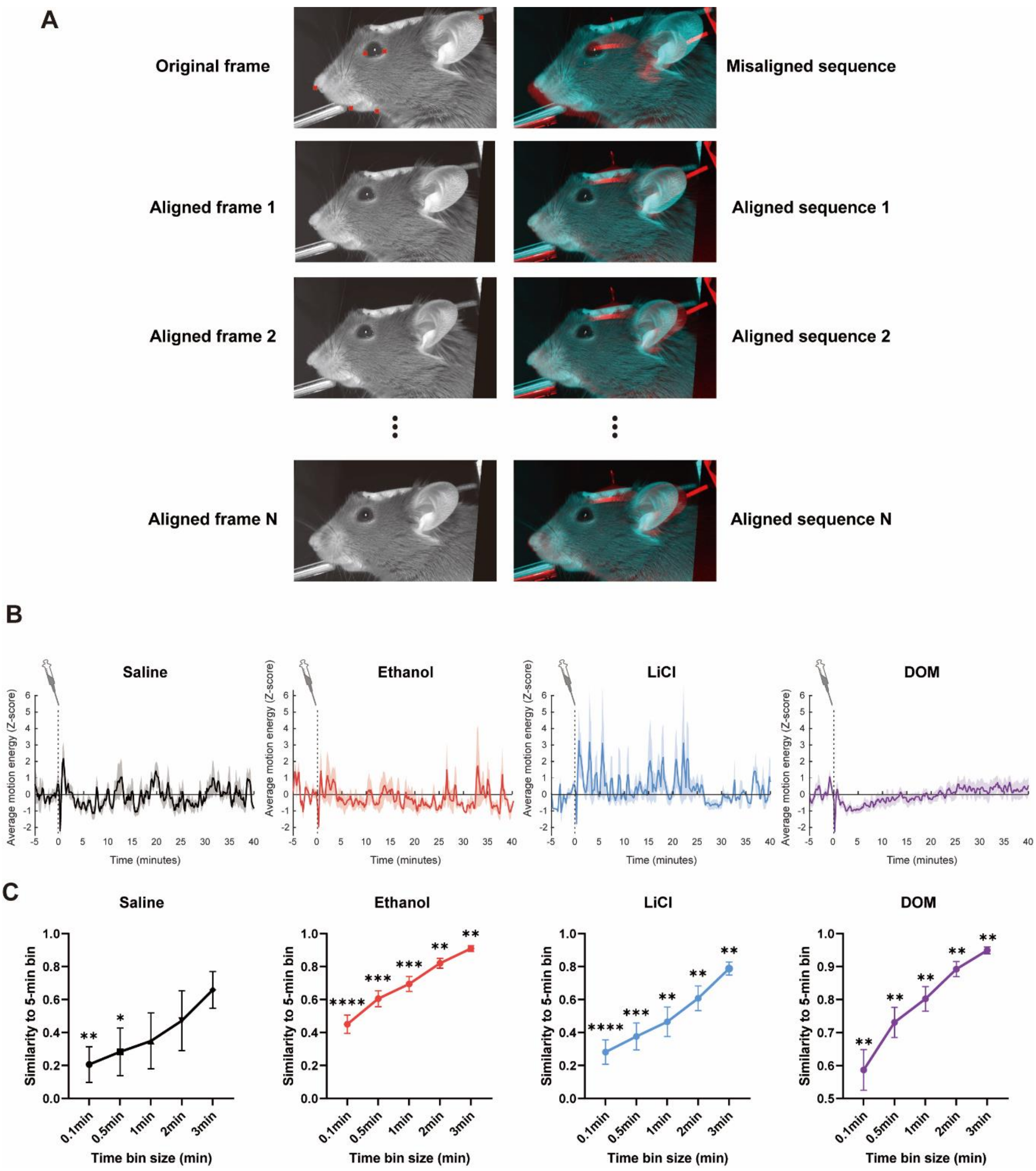

Figure S3. Visual alignment and motion energy analysis of drug-induced facial expressions. (A) The baseline image of an example video with six key facial landmarks (red) and its misalignment with the prototype image (top row), followed by the aligned baseline image (second row), with subsequent frames (2 through n) aligned using the same parameters. (B) Average motion energy of facial expression was computed for the baseline and post-injection videos across four conditions: saline (N = 5 mice, black), ethanol (N = 7 mice, red), LiCl (N = 7 mice, blue), and DOM (N = 7 mice, purple), to assess facial movement changes associated with facial expressions in each condition. Line and shaded area are mean ± SEM. (C) Pearson correlation between facial similarity trajectories calculated using shorter temporal bins (0.1, 0.5, 1, 2, and 3 minutes) and the 5-minute reference bin. Bins shorter than 1 minute reduced stability in saline-injected mice, while LiCl, ethanol, and DOM showed condition-specific divergence at shorter timescales. Data are shown as mean ± SEM across animals. *$p < 0.05$, **$p < 0.01$, ***$p < 0.001$, ****$p < 0.0001$, repeated-measures one-way ANOVA with Geisser–Greenhouse correction followed by Dunnett’s multiple comparisons test.

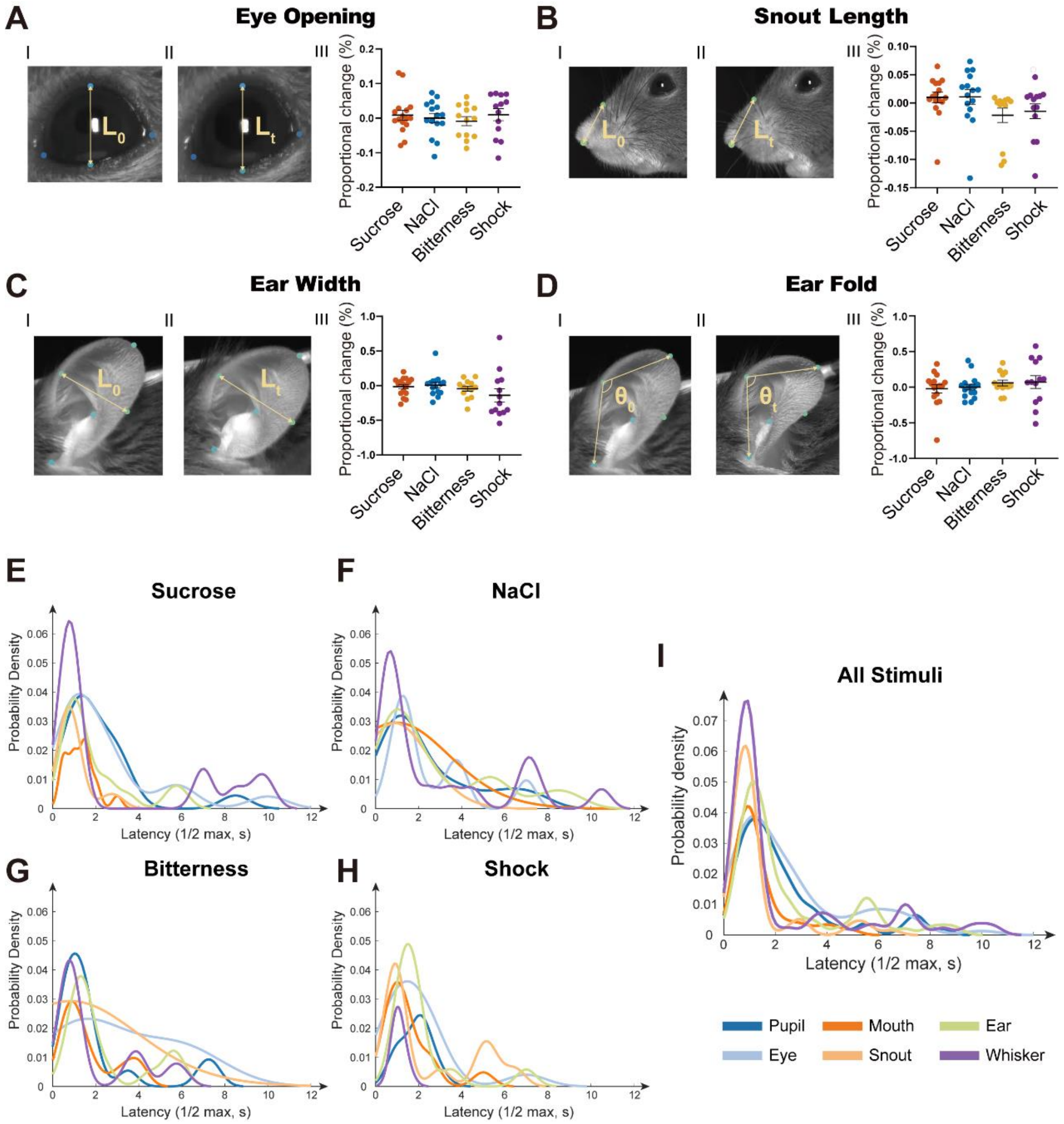

Figure S4. Facial movements during positive and negative stimuli. (A) (Left, I and II) Visualization of identified ear width on the mouse profile. (Right, III) Quantification of peak proportional changes relative to baseline across N = 5 mice (n = 16 trials for sucrose, n = 15 trials for NaCl, n = 13 trials for bitterness, and n = 13 trials for shock), with adjustments made by subtracting the water session peak proportion. No significant differences were observed between conditions ($p > 0.05$). (B) Same as A, but for identified ear fold, illustrating changes in the ear pinna flexion angle. (C) Same as A, but for identified snout length, illustrating the length along the anterior-posterior axis. (D) Same as A, but for identified eye opening, showing the degree of eye lid separation. (E) Probability density estimate of 1/2 latencies of facial movements following sucrose stimulation in mice, based on kernel density estimates. (F) Same as E, but for NaCl stimulation. (G) Same as E, but for bitterness stimulation. (H) Same as E, but for shock stimulation. (I) Probability density estimate of 1/2 latencies of facial movements for all stimuli, based on kernel density estimates. Colors represent different facial regions: dark blue for pupil, light blue for eye, dark orange for mouth, light orange for snout, green for ear, and purple for whisker.

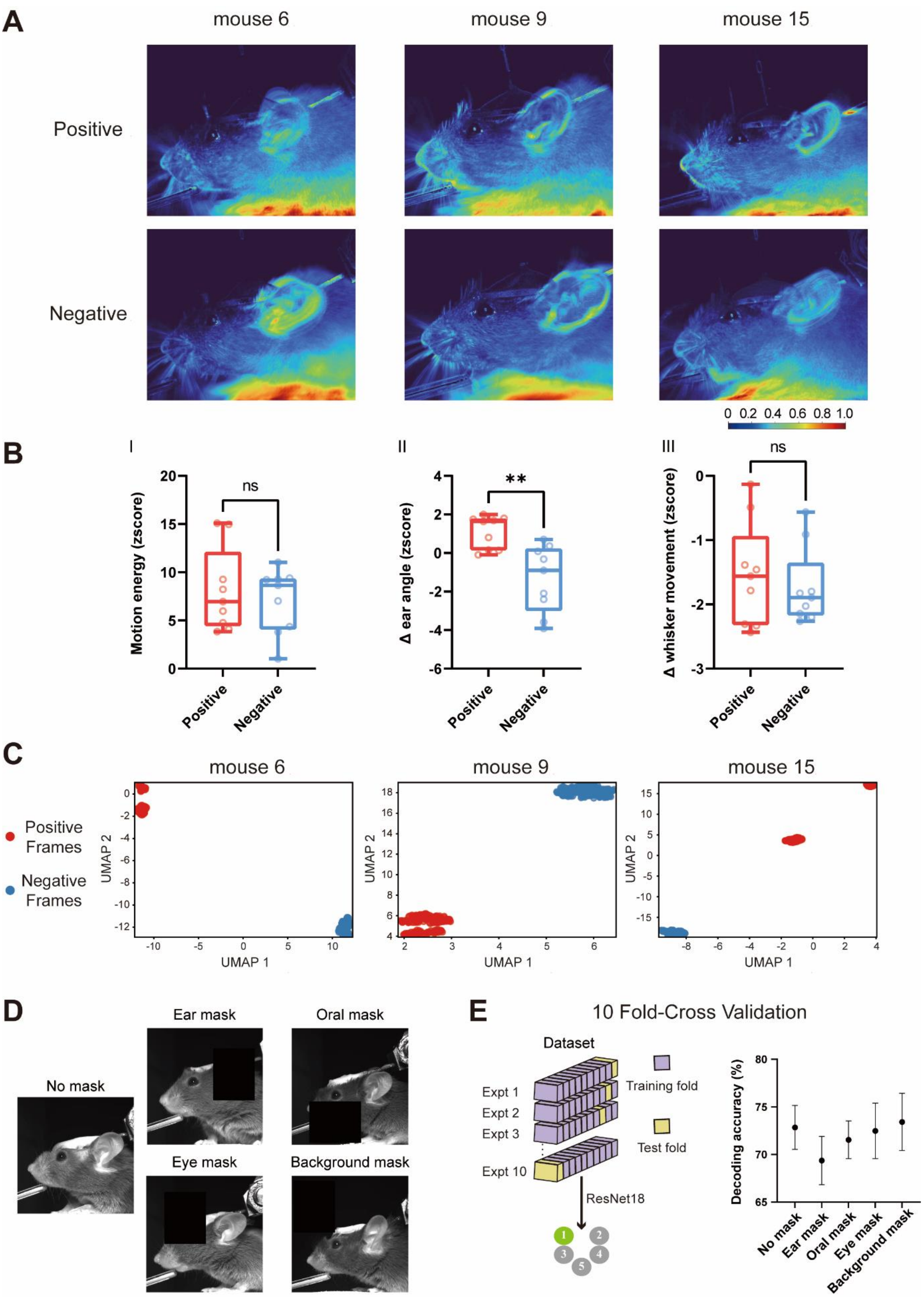

Figure S5. Emotion decoding under high locomotion and region-specific occlusion sensitivity. (A) Motion energy maps from three mice that exhibited running behavior following both positive (sucrose or NaCl) and negative (shock) stimuli. For each mouse, the three trials with the highest motion energy (ME) following positive stimuli and all three shock trials were

included. ME was calculated over the first 2 seconds post-stimulus. (B) UMAP projections of frame-wise HOG features from high-motion trials, with positive (red) and negative (blue) frames shown for each mouse. (C) Quantification of facial motion dynamics based on peak values within the 2-second window post-stimulus. (Left, I) Motion energy (z-scored). (Middle, II) Ear angle change (z-scored). (Right, III) Whisker movement (z-scored). Statistical comparison was performed between positive and negative conditions ($n = 18$ trials from $N = 3$ mice; $^{**}p < 0.01$, unpaired two-tailed t-test). (D) Examples of occlusion masks used in the occlusion sensitivity analysis. Black squares (400×500 pixels) were applied to the ear, oral, eye, or background regions; original (no-mask) input is shown for comparison. (E) Schematic of the 10-fold cross-validation procedure and decoding performance using ResNet18 classifiers under different occlusion conditions. Mean decoding accuracy (mean ± SEM) was highest in the no-mask condition (72.84 ± 2.31%), and dropped most notably when the ear region was occluded (69.36 ± 2.54%), indicating the ear's critical role in emotion classification. Accuracy in the oral (71.54 ± 1.98%), eye (72.47 ± 2.92%), and background (73.41 ± 3.00%) mask conditions remained comparable to the no-mask baseline.

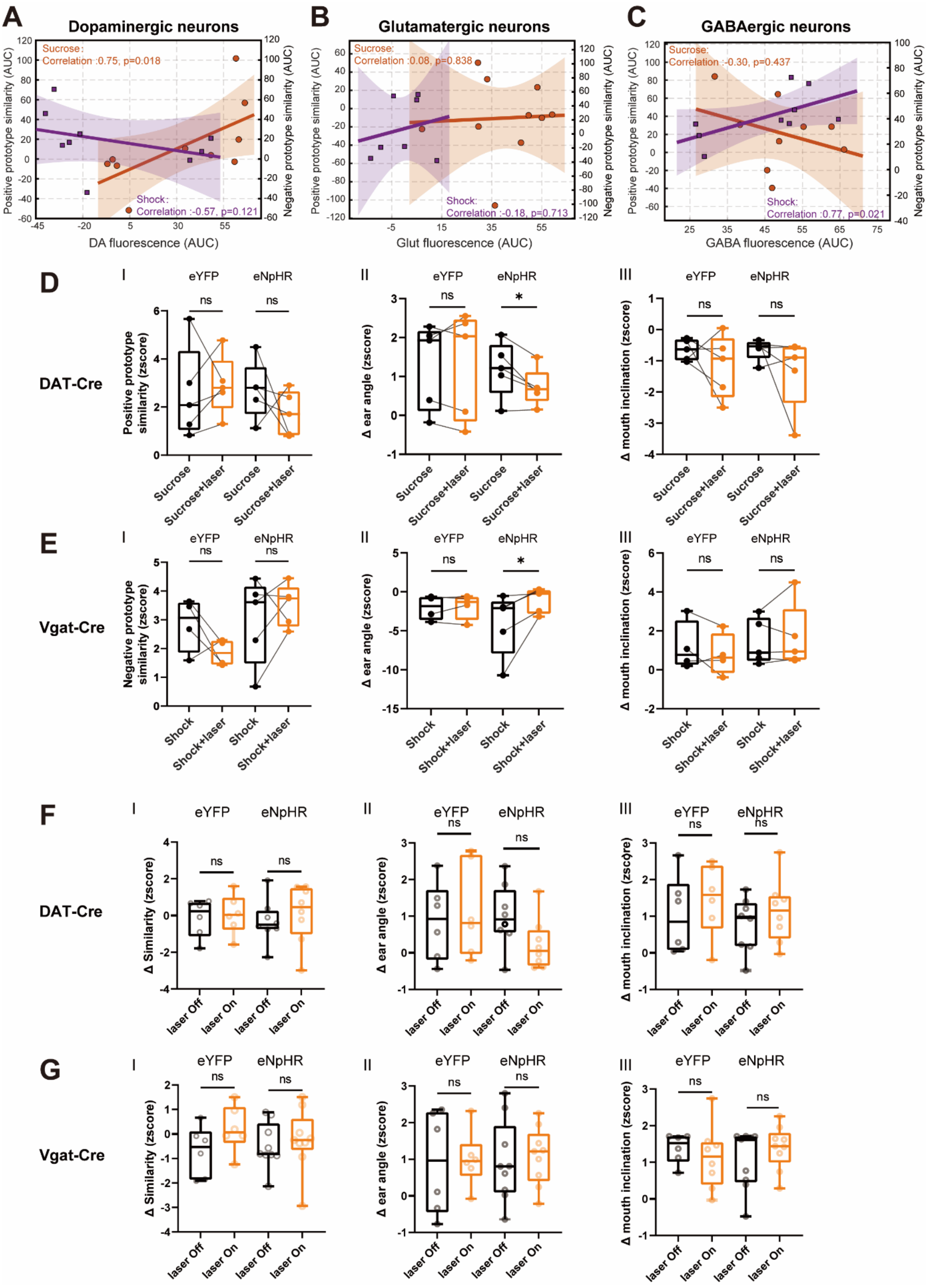

Figure S6. Individual-level analysis of global and regional facial expression changes following optogenetic inhibition (A) Correlation between DA neuron activity and facial similarity during sucrose (orange) and shock (purple) trials. Each dot represents a single trial. Linear regression lines are shown with 95% confidence intervals; Spearman correlation coefficients and p-values are noted. (B) Same as (A), for glutamatergic neurons. (C) Same as (A), for

GABAergic neurons. (D) Effects of optogenetic VTA^{DA} inhibition in DAT-Cre mice, summarized per animal per day (eYFP: N = 5; eNpHR: N = 5). (I) Similarity (z-score) to the positive prototype under sucrose and sucrose+laser conditions. (II) Peak ear angle change (z-score) from baseline. (III) Peak mouth inclination (z-score) from baseline. *$p < 0.05$, paired t-test. (E) Effect of optogenetic VTA^{GABA} inhibition in Vgat-Cre mice under shock or shock+laser conditions (eYFP: N = 4; eNpHR: N = 5). (I) Similarity (z-score) to the negative prototype. (II) Peak ear angle change (z-score) from baseline. (III) Peak mouth inclination (z-score) from baseline. *$p < 0.05$, paired t-test. (F) Effects of laser alone on facial expressions without emotional stimulation in DAT-Cre mice. eYFP group (N = 2 DAT-Cre mice, n = 6 trials for laser-Off, n = 6 trials for laser-On); eNpHR group (N = 3 DAT-Cre mice, n = 8 trials for laser-Off, n = 8 trials for laser-On). (I) Similarity (z-score) to the animal's own pre-laser apex frame. (II) Change in ear angle (z-score) from baseline. (III) Change in mouth inclination (z-score) from baseline. $p > 0.05$, Mann–Whitney test. (G) Same as (F), but for Vgat-Cre mice. eYFP group (N = 2 Vgat-Cre mice, n = 6 trials for laser-Off, n = 6 trials for laser-On); eNpHR group (N = 3 Vgat-Cre mice, n = 9 trials for laser-Off, n = 9 trials for laser-On). Laser was delivered in the absence of sucrose or shock. Each dot represents one trial. (I) Similarity (z-score) to the animal's own pre-laser apex frame. (II) Change in ear angle (z-score) from baseline. (III) Change in mouth inclination (z-score) from baseline. $p > 0.05$, Mann–Whitney test.

Supplementary movies

Movie S1. Facial expression recordings of a head-fixed mouse during intraoral delivery of sucrose (left) or NaCl solution (right) via an intraoral cheek fistula, both inducing positive emotional responses.

Movie S2. Facial expressions of a head-fixed mouse recorded during intraoral delivery of a bitter solution via an intraoral cheek fistula (left) or during tail shock (right), both associated with negative emotional responses.

Movie S3. Facial expression recordings in a DAT-Cre mouse during sucrose delivery with laser-Off (left) and laser-On (right, 20-Hz optical modulation of VTA^{DA} neurons). A marked difference in ear movements is observed when inhibiting DAergic neurons in the VTA: under laser-Off (left), ear angle increases relative to the x-axis, whereas under laser-On (right), ear angle decreases.

Movie S4. Facial expression recordings in a Vgat-Cre mouse during tail shock stimulation with laser-Off (left) and laser-On (right, 20-Hz optical modulation of VTA^{GABA} neurons). A pronounced difference in ear movements is observed when inhibiting GABAergic neurons in the VTA: under laser-Off (left), ear angle decreases relative to the x-axis, while under laser-On (right), ear angle increases.
